# Structural and Functional Properties of Subsidiary Atrial Pacemakers in a Goat Model of Sinus Node Disease

**DOI:** 10.3389/fphys.2021.592229

**Published:** 2021-03-04

**Authors:** Luca Soattin, Zoltan Borbas, Jane Caldwell, Brian Prendergast, Akbar Vohra, Yawer Saeed, Andreas Hoschtitzky, Joseph Yanni, Andrew Atkinson, Sunil Jit Logantha, Balint Borbas, Clifford Garratt, Gwilym Matthew Morris, Halina Dobrzynski

**Affiliations:** ^1^Division of Cardiovascular Sciences, Faculty of Biology, Medicine and Health, Manchester Academic Health Science Centre, University of Manchester, Manchester, United Kingdom; ^2^Manchester Heart Centre, Central Manchester University Foundation Trust, Manchester Academic Health Science Centre, Manchester, United Kingdom; ^3^Liverpool Heart and Chest Hospital, Liverpool, United Kingdom; ^4^Hull University Teaching Hospitals, Hull, United Kingdom; ^5^Hull York Medical School, Hull, United Kingdom; ^6^Department of Medicine, Aga Khan University, Karachi, Pakistan; ^7^Adult Congenital Heart Disease Unit, Manchester Royal Infirmary, Manchester Academic Health Science Centre, Manchester, United Kingdom; ^8^Royal Brompton Hospital, London, United Kingdom; ^9^Imperial College London, London, United Kingdom; ^10^Liverpool Centre for Cardiovascular Sciences, Department of Cardiovascular and Metabolic Medicine, University of Liverpool, Liverpool, United Kingdom; ^11^Department of Anatomy, Jagiellonian University, Krakow, Poland

**Keywords:** sinus node ablation, sinus node disease, subsidiary atrial pacemaker tissue, paranodal area, SND goat model, site of earliest activation, HCN4

## Abstract

**Background:**

The sinoatrial/sinus node (SAN) is the primary pacemaker of the heart. In humans, SAN is surrounded by the paranodal area (PNA). Although the PNA function remains debated, it is thought to act as a subsidiary atrial pacemaker (SAP) tissue and become the dominant pacemaker in the setting of sinus node disease (SND). Large animal models of SND allow characterization of SAP, which might be a target for novel treatment strategies for SAN diseases.

**Methods:**

A goat model of SND was developed (*n* = 10) by epicardially ablating the SAN and validated by mapping of emergent SAP locations through an ablation catheter and surface electrocardiogram (ECG). Structural characterization of the goat SAN and SAP was assessed by histology and immunofluorescence techniques.

**Results:**

When the SAN was ablated, SAPs featured a shortened atrioventricular conduction, consistent with the location in proximity of atrioventricular junction. SAP recovery time showed significant prolongation compared to the SAN recovery time, followed by a decrease over a follow-up of 4 weeks. Like the SAN tissue, the SAP expressed the main isoform of pacemaker hyperpolarization-activated cyclic nucleotide-gated channel 4 (HCN4) and Na^+^/Ca^2+^ exchanger 1 (NCX1) and no high conductance connexin 43 (Cx43). Structural characterization of the right atrium (RA) revealed that the SAN was located at the earliest activation [i.e., at the junction of the superior vena cava (SVC) with the RA] and was surrounded by the paranodal-like tissue, extending down to the inferior vena cava (IVC). Emerged SAPs were localized close to the IVC and within the thick band of the atrial muscle known as the crista terminalis (CT).

**Conclusions:**

SAN ablation resulted in the generation of chronic SAP activity in 60% of treated animals. SAP displayed development over time and was located within the previously discovered PNA in humans, suggesting its role as dominant pacemaker in SND. Therefore, SAP in goat constitutes a promising stable target for electrophysiological modification to construct a fully functioning pacemaker.

## Introduction

Discovered by Martin Flack and Sir Arthur Keith in 1906, the sinoatrial/sinus node (SAN) is the primary pacemaker of the mammalian heart. Structurally and functionally, the SAN has unique properties, different from those of the working myocardium (Keith and Flack, 1907; Linscheid et al., 2019). The mammalian SAN is a crescent-shaped structure located at the junction of the superior vena cava (SVC) and the right atrium (RA) (Boyett et al., 2000; Dobrzynski et al., 2007). The extent of the SAN shows significant variation among mammalian species. In small laboratory animals, the SAN extends inferiorly and occupies the full thickness of the intercaval region adjacent to the crista terminalis (CT), almost reaching the inferior vena cava (IVC). By contrast, in humans, it is confined to a relatively short portion of the cranial intercaval region abutting the CT and separating from the endocardium by a layer of atrial cardiomyocytes (Boyett et al., 2000). In large mammals, the SAN comprises a wide body and a narrower head and tail (He et al., 1991). Recent reconstruction studies of human specimens have shown a much broader SAN, extending down at the posterolateral RA (Boyett et al., 2000; Dobrzynski et al., 2005, 2013; Sanchez-Quintana et al., 2005; Chandler et al., 2011; Atkinson et al., 2013). Various observations, both in animal models and in human patients, suggested the existence of a larger area involved in pacemaking. We have previously reported a detailed anatomical model of the human SAN (Chandler et al., 2011), showing that it begins at the SVC and extends down the CT about one-third of the distance to the IVC. Despite this, the whole CT in humans is commonly associated with physiological and pathophysiological pacemaking (focal atrial tachycardias) (Boineau et al., 1990; Fedorov et al., 2006; Morris et al., 2013, 2019). It is possible that in *Homo sapiens*, the discrepancy between anatomy and function might be explained by a novel paranodal area (PNA), which we have identified to be located within the CT and adjacent to the SAN (Dobrzynski et al., 2005; Chandler et al., 2009, 2011; Morris et al., 2013). Most importantly, the PNA appears to be much more extensive than the conventional SAN tissue (Monfredi et al., 2010; Chandler et al., 2011). Displaying unique features, the PNA might have a pivotal function in generating hierarchical subsidiary atrial pacemaker (SAP) tissue in pathophysiological conditions, such as the sinus node disease (SND) (Monfredi et al., 2010).

SND, sometimes also known as sick sinus syndrome, is an abnormality of action potential generation within the SAN and electrical impulse propagation from the SAN to its surrounding atrial muscle and/or at the interface of the SAN with the working atrial myocardium (Dobrzynski et al., 2013), causing severe sinus bradycardia, sinus pause, sinus arrest, and sinus exit block (Ferrer, 1968; Kaplan et al., 1973; Wu et al., 1992). The prevalence is in one of every 600 cardiac patients over 65 years of age and accounts for ∼20%–50% of pacemaker implants (Benditt et al., 2011; Mond and Proclemer, 2011). In clinical practice, SND commonly affects the elderly population and is often associated with heart failure, diabetes, and atrial fibrillation (Boyett et al., 2009).

Due to its SAN-like properties, PNA might be an interesting target for attempting the generation of biological atrial pacemakers, which can potentially circumvent specific limitations of electronic pacemakers (Rosen et al., 2004). In fact, in both clinical setting and animal studies, it has been demonstrated that, irrespective of the pathological process, once the SAN has failed, SAP tissue takes over as the leading pacemaker (Randall et al., 1978; Rozanski et al., 1983; Littmann et al., 1990; Kalman et al., 1995; Matsuo et al., 2010).

The ion channel expression within the SAN is highly specialized and very different from the atrial muscle (Chandler et al., 2009). Nodal cells and working myocytes present peculiar differences in terms of morphology, ion channel repertoire, and electrophysiological function. While working myocytes have a stable negative resting membrane potential, mainly due to the inward rectifier K^+^ (*I*_*K1*_) current, pacemaker cells show a less negative membrane potential, which arises during the diastole and culminates in a propagating excitation wave (Stanfield et al., 2002; Dhamoon and Jalife, 2005; Choudhury et al., 2015). Although the pacemaking theory remains in part still debated, the SAN automatic electrical activity relies on a combined interaction between specific membrane ion currents (membrane clock) and mechanisms regulating intracellular Ca^2+^-handling (Ca^2+^ clock) (Boyett, 2009; Lakatta and Maltsev, 2012; Choudhury et al., 2015). A key role in pacemaker function is played by the funny (*I*_*f*_) current, named after its unusual feature to conduct an inward depolarizing current when activated on hyperpolarization of the resting membrane potential (Brown et al., 1979). *I*_*f*_ is generated by the hyperpolarization-activated cyclic nucleotide-gated (HCN) channel—predominantly HCN4 (Brown et al., 1979; DiFrancesco, 1995; Azene et al., 2003). The second messenger cyclic adenosine monophosphate (cAMP) directly activates HCN channels at the cytoplasmic side (DiFrancesco and Tortora, 1991). β-Adrenergic stimulation elevates intracellular cAMP, increasing the pacemaker slope and positively affecting chronotropy (Scicchitano et al., 2012). By contrast, muscarinic or purinergic stimulation (adenosine-1 receptor) reduces cAMP availability, generating a profound negative chronotropic effect (Mesirca et al., 2013; Soattin et al., 2017, 2020). Although PNA shows nodal-like features, the ion channel expression in the PNA appears different from both the SAN and the surrounding atrial muscle (Chandler et al., 2009). Some PNA cell clusters express atrial-specific markers, such as connexin-43 (Cx43) and atrial natriuretic peptide (ANP), while others present a nodal-like phenotype. The expression pattern of ion channels in the PNA is intermediate between SAN and atrial muscle; for example, the expression of the cardiac Na^+^ channel (Na_*V*_1.5) and the inward rectifier K^+^ channel (K_*ir*_2.1) is intermediate between that of the SAN and the atrial tissue (Boyett et al., 2000, 2009).

Currently, little is known in humans and in large animal models about the structural and functional properties of PNA and the subsidiary pacemakers. The relatively poor expression of *I*_*K1*_ currents would suggest that these regions may be depolarized, compared to the RA, and therefore capable of pacemaker activity. It is vital to understand their micro-anatomy, histology, electrophysiology, and any possible adaptation the SAP may undergo substituting the SAN. Investigation of the function of the PNA would require the use of a large animal model. Thus, in our study, we developed a goat model of SND by ablating the SAN and characterized the emerging SAP using histology and immunofluorescence methods. Aims of this study were to (1) functionally identify the primary and subsidiary pacemaker regions in the goat RA, (2) structurally characterize these regions, and (3) determine if the PNA exists in this species and evaluate its role as a subsidiary pacemaker in SND.

## Materials and Methods

### Species Used

Adult female goats (*n* = 15) were used. All animal care and usage was according to standards and practices approved by the University of Manchester Animal Welfare and Ethical Review Body and in accordance with the Animals (Scientific Procedures) Act, 1986 (license number: PPL 40/3364). Flow chart of the research protocol is presented in Supplementary Material 1.

### Surgical Technique to Expose the Heart and Gross Anatomical Location of the Sinoatrial Node Region

Anesthesia was induced and maintained with isoflurane (1–3%) in 1:2 mixture of oxygen and nitrous oxide, respectively. The goats were ventilated using positive pressure and placed in the left lateral position. Intraoperative monitoring was continuous throughout the procedure by means of pulse oximetry, non-invasive blood pressure monitoring placed on the tail, and single-lead electrocardiogram (ECG). A lateral thoracotomy was performed and the pericardium opened to expose the sulcus terminalis on the posterolateral RA. At this stage, heart rate was recorded as pre-ablation baseline.

### Epicardial Pacemaker Implantation

An electronic pacemaker (Medtronic Inc.) was implanted to ensure adequate heart rate in the immediate post-operative period. A bipolar epicardial lead was implanted on the RA appendage and the pulse generator inserted in a subcutaneous pocket. The pacemaker was tested *via* a pacing system analyzer (Medtronic Inc.) and was considered satisfactory if pacing threshold was less than 1.5 V and sensed atrial amplitude >2 mV. For assessment of the SAN/SAP recovery time, programmed extra-stimuli were performed *via* the pacemaker during the follow-up period.

### Mapping and Ablation

#### Mapping the Earliest Activation Within the Sinoatrial Node—Procedure I

Epicardial mapping was performed to determine the site of earliest activation (SEA). A quadripolar ablation catheter (3.5-mm tip; electrode spacing 2, 5, and 2 mm; Biosense Webster) was used to map the epicardial RA in a systematic manner guided by a virtual grid constructed using anatomical landmarks to aid reproducibility (Supplementary Material 2). During the mapping process, the RA pacemaker electrode was used as a stable fiducial reference for timing. The earliest local electrogram, preceding the onset of the ECG P-wave, was defined as the SEA. Confirmation of the aforementioned site was obtained by placing the mapping electrode at the putative SEA. Slight shift of the electrode to any direction from the indicated location resulted in a delayed premature local activation time. To display SAN potentials, it was necessary to use a cut-off below 0.5 Hz (Reiffel et al., 1980). In Supplementary Material 3, the RAA bipole, proximal mapping bipole, distal mapping bipole, and the surface ECG are represented. Electrograms were acquired using a filtering and preamplifier system (Digitimer Ltd.) and a PowerLab data acquisition system (AD Instruments) at a sampling rate of 1 kHz. Band-pass filters of 0.3–300, 0.1–30, and 30–300 Hz were applied to the surface ECG, mapping electrode, and RA reference electrode, respectively.

#### Ablation of the Sinoatrial Node

Following identification of the SEA, radiofrequency energy was delivered at the SAN region, utilizing a temperature-controlled, power feedback ablation system (max temperature: 45–50°C, max delivered power: 50–60 W, Boston Scientific Inc.) in experimental goats (*n* = 10). To avoid premature excessive temperature rise at the catheter tip and allow sufficient radiofrequency energy delivery to the tissue, we applied a continuous irrigation at room temperature of 0.9% saline solution at 17–30 ml/min during the ablation procedure. The earliest atrial activation during spontaneous rhythm was remapped after each ablation, the endpoint for radiofrequency application being a conventional decrease in spontaneous heart rate by 50% and/or the emergence of atrioventricular junctional rhythm (Kalman et al., 1995; Supplementary Material 4). Additionally, if the SEA moved away from the accessible epicardial RA (e.g., to the intra-atrial septum or LA), this was an ablation endpoint. In the immediate postoperative period, a pacing rate of 60–80 beats per minute (bpm) was maintained for 24 h. The thoracotomy wound was closed in layers and the animals recovered.

#### Follow-Up Period

After 24–48 h, the electronic pacemakers were reprogrammed to 30 bpm (demand AAI pacing) to allow the emergence of the intrinsic rhythm. After a further 48 h, once an established intrinsic atrial rhythm had emerged, the pacemakers were reprogrammed to sensing-only mode. All experimental goats were followed up for 4 weeks before procedure II. Single-lead ECGs were recorded weekly. Corrected sinus node recovery time (CSNRT) or SAP recovery time (CART) were determined in the resting conscious state.

#### Mapping of the Leading Pacemaker–Procedure II

The perioperative steps leading to exposure of the right atrial free wall were the same as during procedure I. Once visualized, the pacing leads were disconnected from the pacemaker generator and a 5-min ECG was recorded, intrinsic pacemaker recovery time (CSNRT or CART) was determined in the anesthetized state, then mapping of the SEA followed. The site of earliest activation was marked with two fine surgical sutures (2 mm cranial and caudal from SEA). In case the SEA had moved away from the site of the SAN identified in procedure I, then the SAN ablation was deemed successful and the animal was analyzed in the SAP group. By contrast, if the SEA was at the same site as the SAN from procedure I, then the animal was analyzed in the recovered SAN (rSN) group (Supplementary Material 4).

### Methods of Functional Characterization

#### Surface Electrocardiogram

In the experimental group, following the ablation of the SAN (procedure I), ECGs were recorded every 7 days, over the 4-week follow-up. Leads were placed above the bony prominence on the right and left shoulder and the left hip. Bipolar recordings of 30 s were performed consecutively between left to right shoulder, left hip to left shoulder, and left hip to right shoulder (analogous to human ECG leads I, II, and III, respectively). Then, a continuous 5-min recording was created using the left hip–right shoulder bipole (lead II). The leads were connected through a bioamplifier to an analog–digital converter (PowerLab Data acquisition system, ADInstruments) and displayed on a PC using LabChart software (ADInstruments). Beat-to-beat heart rate was calculated in a consecutive 4 min of recording and cleaned from noise/baseline movement with a combination of digital filtering (1–100 Hz band-pass filter) and by rejecting distorted sections using a semi-automatic method (Beat Classifier, LabChart ADInstruments). The mean PR-interval was calculated from 30 consecutive beats.

#### Overdrive Pacing, SAN, and SAP Recovery Time

Sinus node recovery time (SNRT) was assessed by RA stimulation through the epicardial electrodes for 30 s at cycle lengths of 600, 500, 450, 400, and 350 ms. Upon cessation of pacing, the interval between the last paced beat and the first return of the sinus beat (SNRT) was measured three times at each cycle length and averaged. CSNRT was calculated as the difference between SNRT and the base sinus cycle length. The longest averaged CSNRT value was considered the maximal CSNRT. The corrected recovery time of the subsidiary pacemaker (CART) was measured using the method described above. CART was assessed every week in the post-ablation period in the conscious animals and during the second SAP mapping procedure under general anesthesia (Narula et al., 1972).

#### Monitoring the Mean Heart Rate

An implantable loop recorder (Reveal Dx, Medtronic Inc.) was inserted at the end of procedure I in three goats. On the opposite ends of the recorder, there were two electrodes acting as a bipolar recorder, capable of automatically recording a 1-min ECG, when heart rate was either lower than 30 bpm or in the presence of a pause longer than 2 s. The recorder was inserted in a superficial subcutaneous pocket overlying the heart.

### Processing of the Right Atrial Tissue With Primary and Subsidiary Atrial Pacemaker Regions

#### Harvesting and Dissection of the Heart

**FIGURE 1:**
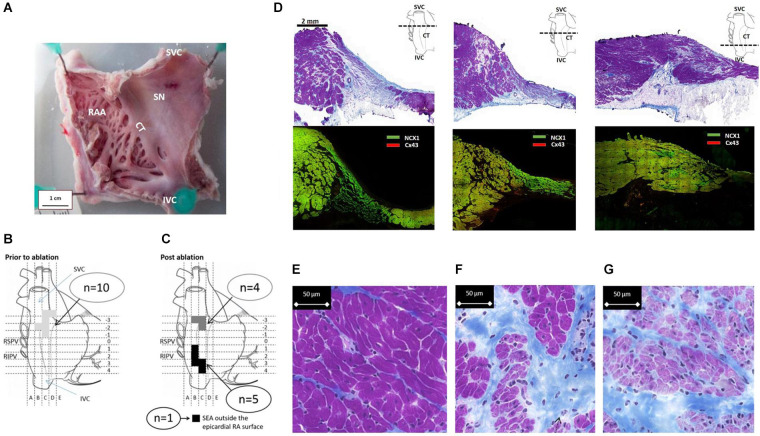
Functional evaluation of the SAP region and the SAN in goats. **(A)** Fresh preparation of the posterior wall of the right atrium (RA) from a control goat. CT, crista terminalis; SAN, sinus node; IVC, inferior vena cava; SVC, superior vena cava. Schematic diagrams **(B,C)** depict the locations of the site of earliest activation during mapping and ablation experiments. **(B)** Location of the site of earliest activation (SEA) prior to ablation (*n* = 10) is represented by the shaded area (light gray). **(C)** After ablation, the shaded squares correspond to regions of the RA where each individual SEA shifted to. Two well-separated areas are defined: one cluster is close to the IVC/RA junction (black), while the second cluster remained close to the RA/SVC junction (dark gray); *n* = 6 are shown; one SEA was outside the accessible epicardial RA surface and is therefore presumed to be either on the interatrial septum or left atrium. **(D)** Cross-sections of the SAN and PNA, taken perpendicular to the CT at the levels displayed by the dashed line in the schematic. The top row shows Masson’s trichrome (connective tissue stains royal blue, the cytoplasm pink, and the nuclei dark blue). The area of the SAN is identifiable by its pale appearance. The SAN occupies the full thickness of the intercaval region in the superior sections. The PNA appears as a loosely packed bundle, which is localized between the SAN and the working atrial myocardium. The bottom panels in panel **(D)** shows adjacent sections double labeled for NCX1 protein (green) and Cx43 protein (red); SAN tissue is characterized by the presence of NCX1 but the absence of Cx43 and thus appears bight green on these images due to the absence of red signal from Cx43. Regions of nodal tissue can be identified in all sections including the most inferior part of tissue. **(E–G)** High-magnification images of Masson’s trichrome-stained atrium **(E)**, PNA **(F)**, and SAN **(G)**. The SAN cells are smaller, paler (stained purple), and embedded in a rich connective tissue (royal blue) compared to the atrial myocardial cells.

After *in vivo* experiments (as shown in Supplementary Material 1), the goats were again anesthetized with isoflurane (1–3%) in 1:2 mixture of oxygen and nitrous oxide. The heart was exposed through the previous lateral thoracotomy. Following this, the goats were humanely euthanized with a lethal dose of intravenous pentobarbital injection, in accordance with the Home Office Animals (Scientific Procedures) Act, 1986. Immediately after cessation of cardiac activity, the heart, along with the proximal portions of the IVC and SVC, was removed and placed in ice-cold Tyrode solution. The atrial region, where the SAN (ablated and non-ablated) and SAP regions were identified as described above, was dissected. The final preparation contained the pectinate muscle and CT of the RA and intercaval region proximal segments of the SVC and IVC (Figure 1A). Before freezing the tissue, the preparations (due to their size) were divided perpendicularly to the CT into two segments for cryosectioning. Preparations were frozen by submerging in isopentane (Sigma-Aldrich), cooled to −50°C in liquid nitrogen, and tissue segments were stored at −80°C.

#### Cryosectioning

The SAN/PNA/SAP preparations were mounted and sectioned perpendicular to the CT. For each millimeter of the tissue, four consecutive sections at 10 μm (for histology) and 20 consecutive sections at 20 μm thickness (for immunofluorescence staining) were collected and placed in pairs on Superfrost Plus Microscope Slides. Slides were then stored at −80°C.

#### Histology and Light Microscopy

Masson’s trichrome histology staining was used as previously described (Chandler et al., 2009, 2011). For detailed analysis of cell morphology within the RA, PNA, SAN, and SAP, light microscopy was used with a motorized stage (Zeiss) utilizing a high-power magnification (× 63) immersion objective. To delineate the boundaries of the SAN (with PNA region) or SAP regions within the RA, a panoramic automated light microscope was used for this purpose creating a single digital slide at ×20 magnification covering the whole section (3DHISTECH).

#### Immunofluorescence and Microscopy

Immunofluorescence (IF) experiments were carried out according to an established protocol previously described (Chandler et al., 2009; Yanni et al., 2010; Morris et al., 2013). Slides were removed from the freezer and tissue sections were demarcated with a hydrophobic PAP pen (Sigma-Aldrich). To fix the tissue, sections were then immersed in 10% buffered formalin (Sigma-Aldrich) for 30 min and rinsed three times in 10 mM phosphate buffered saline (PBS). Permeabilization of the cell membrane was achieved with detergent (0.1% Triton-X100, Sigma-Aldrich), followed by repeated rinses in PBS. To reduce non-specific binding, preparations were treated with 1% bovine serum albumin (BSA). Primary antibodies (diluted in a range of 1:50–1:800, Supplementary Table 1) were applied within the boundaries of the circles created by the PAP pen and incubated overnight at 4°C. Following the incubation period, antibodies were removed by washing the slides in PBS three times before and after the secondary antibodies were applied (Supplementary Table 2) and incubated for 90 min at room temperature. FITC-conjugated antibodies were diluted to 1:100 and Cy3-conjugated ones to 1:400 (Supplementary Table 3). Finally, the IF-labeled slides were mounted in VECTASHIELD anti-fade medium (Vector Labs). To avoid fading of the fluorochrome, the slides were kept in a refrigerator at 4°C in the dark after staining. A laser confocal scanning microscope (Zeiss LSM5) and a scanner (with an epifluorescent objective, 3DHISTECH) were used to visualize the immunolabeled sections at a magnification range between ×10 and ×63. Pairs of slides prepared for immunofluorescence and light microscopy histology were compared.

To avoid differences in intensity and specificity of staining, sections were of equal thickness and were stained simultaneously using the same batch of primary and secondary antibodies. Immunolabeled slides were imaged within 1 day, keeping a constant pinhole size and laser energy output (Chandler et al., 2009).

#### Intracellular Action Potential Recordings

Sharp microelectrodes were used to record intracellular action potentials in one freshly isolated right atrial tissue preparation as previously reported (Logantha et al., 2019). Borosilicate glass capillaries were filled with KCl (3 M) and coupled to an Ag–AgCl holder (Model E45P-M15N, Harvard Apparatus, United Kingdom). Resistance developed by the microelectrode ranged between 20 and 40 MΩ. A disk electrode made of Ag–AgCl (Model E242, Harvard Apparatus, United Kingdom) placed in the bath functioned as ground return. Atrial tissue was superfused with Tyrode buffer solution with 0.05 μM isoprenaline (Sigma-Aldrich) at 37°C, bubbled with a mixture of oxygen/carbon dioxide (95:5).

### Data and Statistical Analysis

Data are reported as the mean ± SEM. Statistical significance was evaluated using one-way ANOVA and/or unpaired *T*-test. In all statistical analyses, *p*-values < 0.05 were considered statistically significant and indicated with an asterisk (^∗^). Illustrations in Supplementary Material were created with BioRender^1^.

## Results

### Sinus Node Ablation and Location of the Subsidiary Atrial Pacemaker Region

In all animals (*n* = 10), the SEA was found in a 1.5-cm^2^ area at the SVC/RA junction (Figure 1B), where typically the SAN tissue localizes (Figure 1D). The endpoint of minimum heart rate 50% reduction and a shift of pacemaker away from the initial SEA were acutely achieved in all animals. There were 40 ± 27 (range: 18–77 min) ablation lesions created and the mean time of power delivery was 27 ± 20 min (range: 5–68 min). After a follow-up of 4 weeks, the location of the leading pacemaker was mapped during the surgical procedure II and the SEA was accurately located in nine out of 10 goats (Figure 1C); in one goat, the SEA was not found at the accessible epicardium. In five goats, the SEA locations clustered along the caudal portion of the CT adjacent to the IVC. In four goats, the SEA had not changed location from the pre-ablation SAN pacemaker region. There was a trend to an inverse correlation between time of applied ablation power and the success of the ablation procedure (*p* = 0.09, Supplementary Material 5). Histological sections confirmed the ablation of the SAN region in experimental animals. By contrast, the caudal regions were not ablated and contained nodal-like loosely packed cells within the CT (Supplementary Material 6).

### Histological and Immunofluorescence Features of the Sinus Node

**FIGURE 2:**
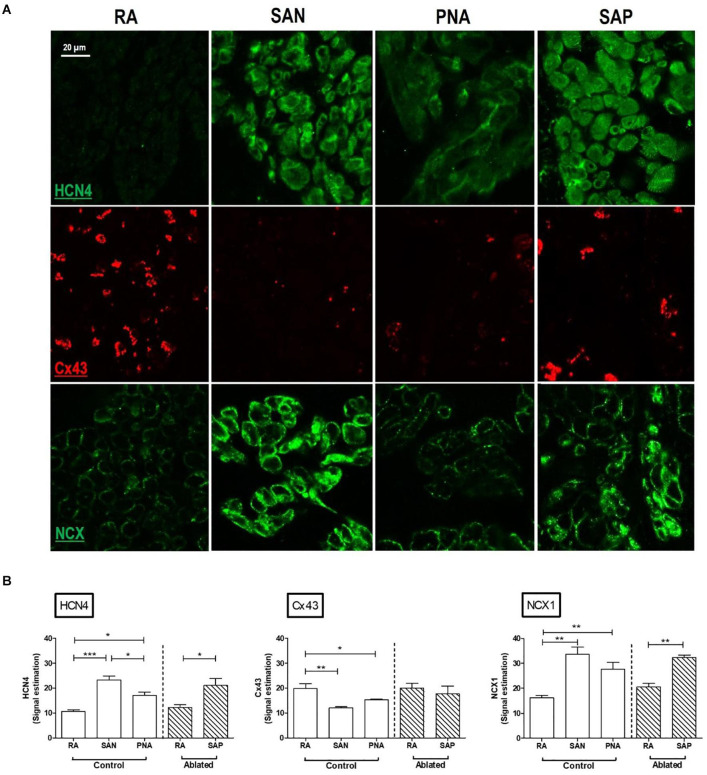
Immunofluorescence comparison of the right atrium with the pacemaker regions. SAN, sinus node; PNA, paranodal area; SAP, subsidiary atrial pacemaker. **(A)** Immunofluorescence (IF) signal is shown for HCN4 protein in green (top rows), Cx43 protein in red (middle rows), and NCX1 protein in green (bottom rows). Sections were taken from RA, SAN, PNA, and SAP. **(B)** Semi-quantification of immunofluorescence signal intensities is shown, allowing comparison among RA, SAN, and PNA in control animals and the RA and SAP (leading pacemaker after ablation) in ablated animals *p < 0.05, **p < 0.01, and ***p < 0.001.

Analysis of serial histological sections showed that the SAN tissue occupies the full thickness of the intercaval region from the endocardium and epicardium abutting the CT (Figure 1D and Supplementary Material 7). Three Masson’s trichrome-stained tissue sections (Figure 1D) are from the superior, mid, and inferior parts of the preparation shown in Figure 1A. In this species, the SAN is composed of the body (left panel), tail (middle panel), and the nodal-like bundles (right panel) within the PNA. Masson’s trichrome-stained tissue section revealed pacemaker tissue, which stains paler when compared with working myocardium (see Figure 1A). The SAN appeared lightly stained with Masson’s trichrome compared to the surrounding atrial muscle and is embedded in a network of blue connective tissue. This is more obvious at high-power magnification (Figures 1E–G). Cell diameter within the SAN was significantly smaller than in the surrounding atrial myocardium (SAN cells 10.1 ± 0.7 μm vs. RA cells 13.8 ± 0.6 μm, *p* < 0.05). Double labeling of the adjacent histological sections for Cx43 and NCX1 by IF technique confirmed the accuracy of the histological location of the SAN, showing a low expression of Cx43 and high expression of NCX1 compared to the atrial muscle (Supplementary Material 8). The SAN and PNA, but not the atrial muscle, also expressed HCN4 and reverse was observed for Cx43 (Figure 2). Similar histological and IF pattern was observed in *n* = 4 tissues (Figures 1, 2 and Supplementary Table 4). Semi-quantification of IF images (Figure 2A) showed that there was significantly more expression of HCN4 (as well as NCX1) in the SAN and PNA vs. RA, and the reverse is true for Cx43 expression (Figure 2B).

### Histological and Immunofluorescence Features of the SAP Tissue: The “Witch” Fingers

**FIGURE 3:**
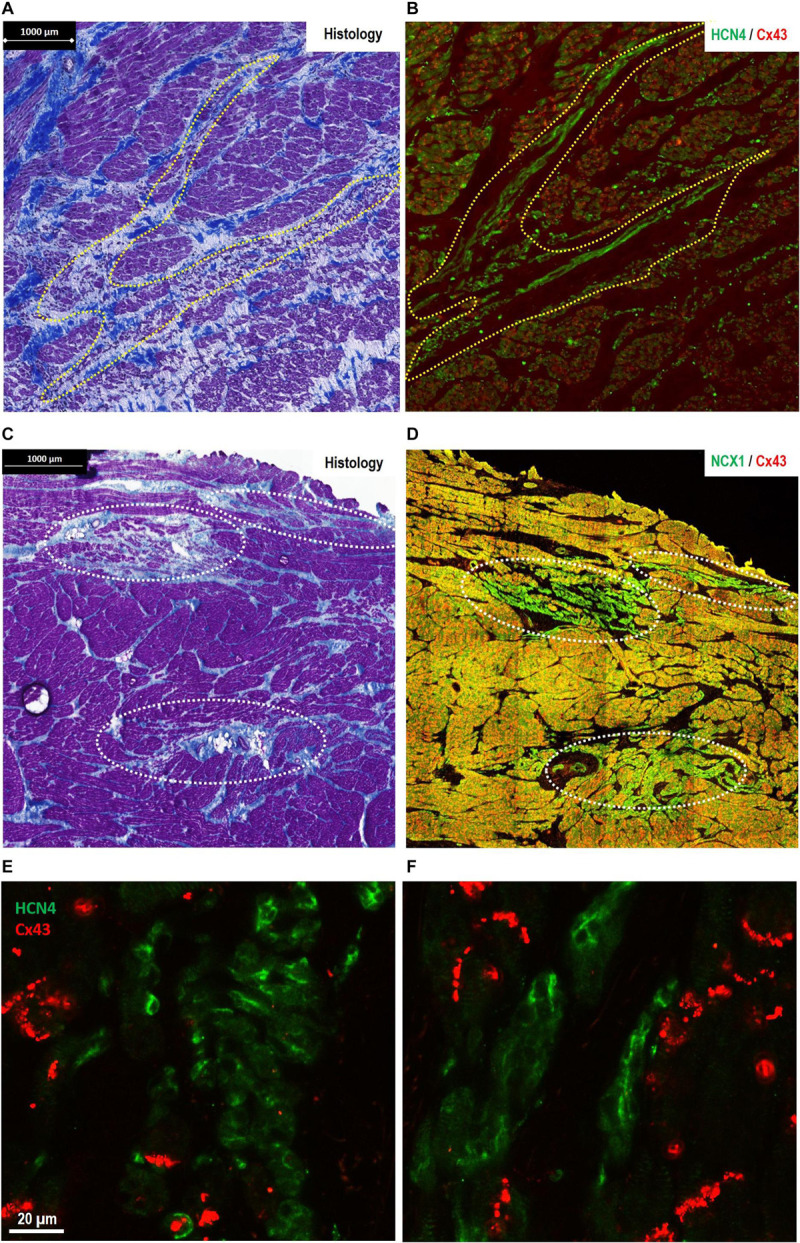
Morphological features of pacemaker/sinus node (SAN)-like tissue in regions other than the primary SAN [where the site of earliest activation (SEA) is detected in healthy hearts]. **(A,B)** The finger-like projections of pacemaker-like tissue in the paranodal area (PNA)—“witch fingers.” **(A)** Histology (Masson’s trichrome) section of the PNA from a control goat (outlined in yellow, A). **(B)** Location of nodal-like cells are confirmed by immunofluorescence labeling of an adjacent section double labeled for Cx43 (red) and HCN4 (green). The nodal-like “witch” fingers in the PNA are predominantly HCN4^+^/Cx43^–^. **(C,D)** Islands of pacemaker-like cells within the subsidiary atrial pacemaker (SAP) regions—“lonely islands.” **(C)** Masson’s trichrome staining showed that cells with nodal-like phenotype in the SAP region formed well-defined islands in sections taken from the CT. **(D)** These “lonely islands” are confirmed to have “nodal”-like phenotype by IF labeling of an adjacent section Cx43 (red) and NCX1 (green). The nodal-like island SAP are predominantly NCX1^+^/Cx43^−^. Corresponding high-power images of sections stained for HCN4/Cx43 are shown for SAP islands in panel **(E)** and “witch fingers” in panel **(F)** demonstrating clusters of HCN4^+^/Cx43^−^ pacemaker-like cells (green) among the Cx43^+^ atrial myocytes (red).

In control goats, the SAP tissue (similar to the previously described PNA in human) was detected at the interface of the SAN with the RA. As shown in Figure 1D, the PNA in the goats localized within the CT close to the SAN and appeared as an extensive structure extending the length of the pacemaker complex caudally as well as dorsally. Masson’s trichrome showed inter-digitations or “witch” fingers of nodal-like cells with atrial cells in histological images (Figure 3A). IF on the sister section confirmed that these “witch” fingers were indeed of nodal nature by being Cx43 negative and HCN4/NCX1 positive (Figures 3B,F).

**FIGURE 4:**
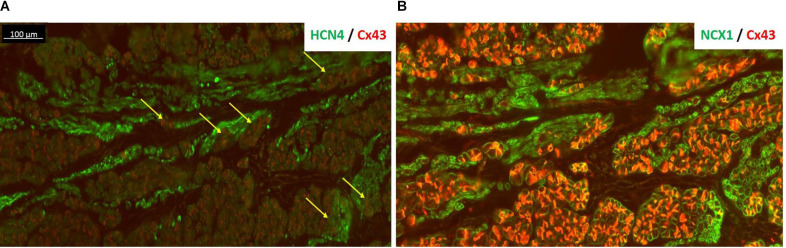
HCN4 is present in the subsidiary atrial pacemaker (SAP) tissue. **(A)** The SAP tissue was double labeled for HCN4 (green) and Cx43 (red) proteins. The nodal-like cells are very distinct within the atrial muscle cells showing a strong HCN4 signal. The very tight contact among SAP and atrial cells is more pronounced in regions shown by the yellow arrows. **(B)** An adjacent section of SAP tissue was double labeled for NCX1 (green) and Cx43 (red). NCX1 labels all myocytes and highlights the region of nodal tissue, which has no red signal for Cx43. Note that in panel **(B)**, Cx43 signal is brighter than panel **(A)** due to the use of a different antibody [rabbit in panel **(B)** rather than mouse in panel **(A)**].

Figure 4 also shows the “witch” fingers within the CT. Here, the SAP tissue was double-labeled for HCN4 (green) and Cx43 (red) proteins (Figure 4A) or NCX1 (red) and Cx43 (green) proteins (Figure 4B). The nodal-like cells are positive for HCN4 and NCX1, but negative for Cx43. However, the “witch” fingers were embedded within the Cx43-positive atrial tissue and this is why there is no difference in Cx43 expression between the RA and SAP in ablated animals as shown in Figure 2B. HCN4 and NCX1 are significantly higher (Figure 2B).

### Histological and Immunofluorescence Features of the SAP Tissue: The “Lonely” Islands

In the successful ablation group, the location of the SAP was found in the caudal half of the intercaval region adjacent to the CT. Histological features of the SAP showed similarities with the PNA. Embedded in a large mass of atrial myocardium there were thin “lonely” islands of nodal-like cells and an intimate large-surface connection with the surrounding atrial myocytes recognizable at the level of the mapped SEA (Figures 3C,D). With IF technique, nodal-like islands showed HCN4/NCX1-positive labeling within Cx43/NCX1-positive labeling within the surrounding myocardium (Figures 3D,E).

### Features on Recovered SAN Group

**FIGURE 5:**
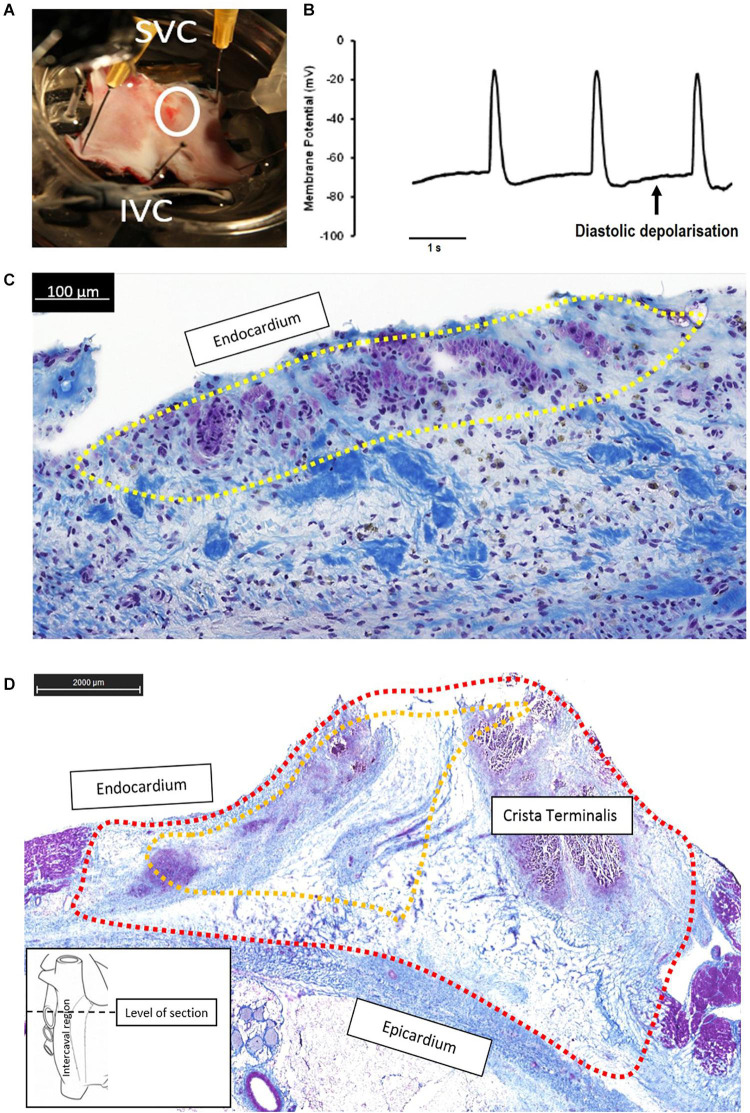
Continued pacemaker function in the failed ablation goat (recovered sinus node, rSN) may be due to small areas of surviving “peripheral” sinus node (SAN) cells that are able to generate diastolic depolarization and thus pacemaker function. **(A)** Right atrial preparation taken from a goat in the rSN group. The ablated area is shown by the white circle, and the superior and inferior vena cava (SVC, IVC) are annotated for orientation. *In vivo* epicardial mapping had confirmed that the leading pacemaker was in this region indicating recovery of SAN function. **(B)** Spontaneous action potentials showing diastolic depolarization was recorded by means of sharp microelectrodes in the region indicated by the white circle in panel **(A)**, the apparently ablated SAN. **(C)** The corresponding histological section (Masson’s trichrome) from this region confirms a thin endocardial strip of surviving nodal cells (shown encircled by yellow dotted line) surrounded by a region of fibrosis (blue) and dark nuclei presumably of necrotic cells. In this example, the application of endocardial radiofrequency energy was not able to generate a full-thickness ablation lesion, meaning a small region of endocardial cells were spared. **(D)** Histology section (Masson’s trichrome) at the level of the ablated SAN from a successfully ablated animal (subsidiary atrial pacemaker, SAP group). The ablated region is circled by the red dashed line and incorporates a full-thickness radiofrequency ablation lesion extending from the epicardium to the endocardium and encompasses the whole area where the SAN would be expected to be found (yellow dashed line). The section shows dense fibrosis (blue staining) with disrupted myocardium (purple staining) and no identifiable clusters of nodal cells.

The failed ablated SAN tissue was used for electrophysiological investigation (Figure 5A). Glass microelectrode analysis of one fresh rSN preparation showed spontaneous diastolic depolarization in post-ablated regions, consistent with its pacemaker activity (Figure 5B). Histological analysis confirmed the presence of surviving *clusters* enriched of nodal cells close to the endocardium (Figure 5C). Interesting to notice that despite the visible lesions to the SAN due to ablation, a relatively small nodal tissue appeared working and able to maintain a pacemaker function. On the other hand, in the histological section of successfully ablated animals (Figure 5D and Supplementary Material 6), the ablated region incorporates a full-thickness radiofrequency ablation lesion extending from the epicardium to endocardium (Figure 5D).

**FIGURE 6:**
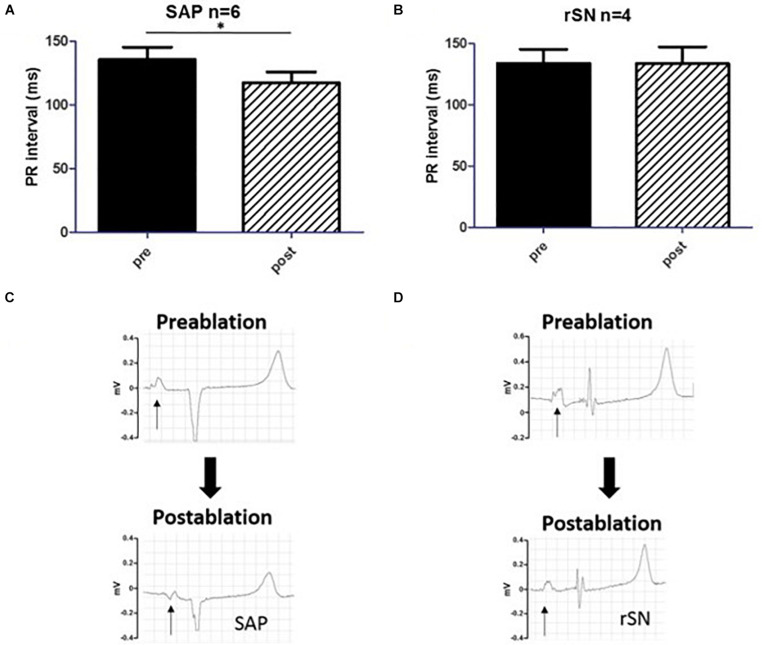
PR-interval and P-wave morphology. **(A,B)** The comparison of PR-intervals pre and post the ablation procedure, respectively. **(A)** The PR-interval shortened significantly in the subsidiary atrial pacemaker (SAP) group (pre vs. post, *p* < 0.05, *n* = 6). **(B)** By contrast, no difference was detectable among the experimental animals [recovered sinus node (rSN), *n* = 4] in which ablation was not successful. **(C,D)** P-wave morphology change before and after the ablation procedure in SAP and rSN goats, respectively. **(C)** Surface electrocardiogram (ECG) shows a significant inversion of the first portion of the P-wave (arrow) post-ablation in the SAP. **(D)** No such shift and P-wave morphology change are observed in rSN goats. *P < 0.05.

By averaging *n* = 30 consecutive beats, PR-intervals were measured in each experiment before (pre) and after (post) the ablation procedure (Figure 6A). In the goats where the SEA was mapped in proximity of IVC (SAP group), PR-interval was significantly reduced after ablation (pre 136 ± 24 ms vs. post 117 ± 21 ms, *n* = 6, *p* < 0.05). This was consistent with the anatomically different location of the SAP compared to the SAN. By contrast, no difference was detected among goats in which ablation had failed (rSN group, Figure 6B). These data confirmed the assumption that the closer the dominant pacemaker is to the AV node, the shorter the PR-interval will become. In three out of the six SAP goats, the first right atrial component of the P-wave morphology appeared inverted in a caudo-cranial fashion (Figure 6C). On the contrary, no change in P-wave morphology was assessed in the rSN group (Figure 6D).

### Heart Rate During Follow-Up

**FIGURE 7:**
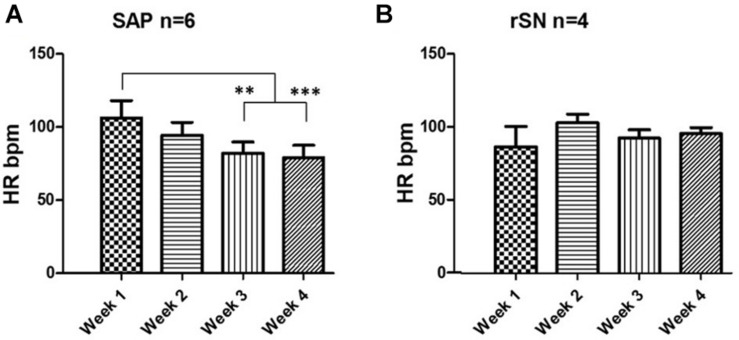
Heart rate change during long-term follow-up. **(A)** Telemetry heart rate (HR) monitoring indicates a significant HR drop in the third and fourth week of the post-ablation in the goats where emergence of subsidiary atrial pacemaker (SAP) was found (repeated measures ANOVA, ^∗∗^*P* < 0.001 and ^∗∗∗^*P* < 0.0001, respectively). **(B)** No significant change was observed in the goats with recovery of SAN function (rSN).

Following SAN ablation, the mean heart rate (HR) was reduced by ∼50% in the majority of the experiments. Thereafter, atrial rhythm quickly recovered and no statistically significant difference was observed between the pre-ablation and post-ablation state (Supplementary Material 9). Among SAP (*n* = 6) and rSN (*n* = 4) goats, no significant difference in HR was observed. However, HR was significantly reduced from week 1 toward week 4 in SAP goats (Figure 7A). By contrast, no significant changes were detected in rSN goats’ HR (Figure 7B).

### Recovery Time and Long-Term Events

**FIGURE 8:**
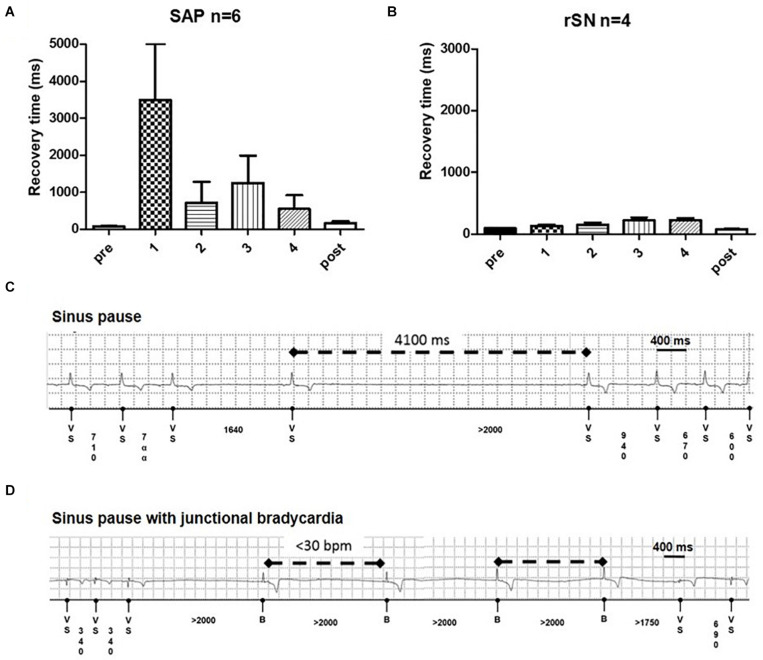
Recovery time and junctional events on long term. **(A)** Subsidiary atrial pacemaker (SAP) group (*n* = 6) shows a profound recovery time (CART) prolongation in the first week of follow-up (*p = 0.04). **(B)** No such prolongation is detectable in the recovered sinus node (rSN) group (*n* = 4). From the second week on, the CART values diminished and became no longer significantly longer than sinus node recovery time (SNRT) at baseline. Examples of **(C)** profound sinus pause and **(D)** sinus pause with junctional bradycardia in ablated goats.

Overdrive pacing from the RA briefly suppressed the native pacemaker. Corrected SAP recovery time (CART) and corrected SAN recovery time (CSNRT) were calculated both intraoperatively and weekly in the 4-week follow-up period. In the first week, CART became more prolonged in all SAP goats (Figure 8A). By contrast, CSNRT was very short (84.3 ± 16.6 ms) at baseline (Figure 8B). CART prolongation trend normalized over 2–4 weeks follow-up. Among SAP goats with implanted loop recorders, pauses up to 6 s (Figure 8C) and multiple episodes of significant junctional bradycardia (<30 bpm) were detected (Figure 8D). These abnormalities were only in the first 3 weeks after ablation, with no pauses or bradycardia <30 bpm detected in the 4th week (data not shown).

## Discussion

The majority of SND is idiopathic, and despite the frequency that the condition occurs, the underlying primary pathophysiology of SND is not well known (Kalman and Morris, 2018). Possible pathological processes include fibrosis and degeneration (Morris and Kalman, 2014; Csepe et al., 2015), but recent data have suggested a prominent role of electrical remodeling, *via* changes in ion channel expression and function (D’Souza et al., 2013; Morris and Kalman, 2014). Due to the difficulty of studying disease in humans, large animal models may help translation of fundamental research findings to clinical benefit (Clauss et al., 2019). In this study, we described the SAN morphology (using a traditional histology method) in the goat, which is a common model for atrial arrhythmia. We also present data regarding the physiology and subsidiary pacemaker areas of a large animal model of SND and provide insight into the well-described challenges of sinus node ablation in humans (Marrouche et al., 2002).

### The Sinus Node and Paranodal Area of the Goat

Though the anatomically determined SAN in humans is described as a compact node at the junction of the SVC and the RA, the leading cardiac pacemaker can be found at many points along the CT, from the SVC to the IVC (Boineau et al., 1988). Animal studies proposed that this may be due to leading pacemaker shift within the SAN itself (Boyett et al., 2000), but contemporary optical mapping studies of human heart suggest that this may be at least in part due to discrete exit pathways from the SAN along the CT (Fedorov et al., 2010). It has also been shown that when the SAN is quiescent or removed, SAP tissue outside of the SAN region can act as the primary cardiac pacemaker (Morris et al., 2013).

Histological and molecular investigations have shown that the functional human SAN is extensive, spanning one-third of the distance to the IVC, and is surrounded by paranodal cells, which constitute the PNA, a distinct micro-anatomical structure extending toward the IVC. The PNA occupies a larger area than the anatomic SAN and could function as a subsidiary pacemaker in pathophysiological conditions (Chandler et al., 2009, 2011). Although the goat is a well-established and widely used model of atrial arrhythmia (Wijffels et al., 1995; van Hunnik et al., 2018), the structure and organization of the SAN in goats are not well known. Data presented here show that the SAN was located in the intercaval region occupying its full thickness from the endocardium to the epicardium and running parallel to the cranial half of the CT as in other smaller animals, like rabbits (Coppen et al., 1999; Zhang et al., 2003). As previously described, the SAN in the goat consisted of small and lightly stained cells, when compared to atrial myocytes. Moreover, nodal cells were embedded in a network of connective tissue. In fact, histological observations have shown that the SAN presents a higher collagen content than the surrounding working myocardium (Csepe et al., 2016). As in other species, all nodal cells expressed high levels of HCN4, which is an important component of the pacemaking *I*_*f*_ current (Li et al., 2015). In addition, goat SAN cells expressed NCX1 but not Cx43. It is interesting to notice that the PNA in goats, which we have identified as a possible SAP tissue, resembles the same anatomical features as in humans. In fact, the PNA/SAP tissue arises on the endocardial side of the CT, extending caudally beyond the SAN as in the human heart (Chandler et al., 2009, 2011). Moreover, the PNA in the goat heart within the CT contained nodal cells (HCN4- and NCX1-positive and Cx43-negative cells) and atrial myocytes (HCN4-negative and NCX1- and Cx43-positive cells). These data suggest that goats and humans share a similar molecular architecture of the PNA and SAP (Chandler et al., 2009). Finally, whereas the SAN was located close to the SVC, the PNA extended along the entire length of the CT from the SVC to the IVC. Interestingly, we also observed nodal-like islands and inter-digitations within the CT, expressing HCN4- and NCX1-positive cells.

### Subsidiary Pacemaker Regions

In order to investigate the primary and subsidiary pacemakers in goats, in this work for the first time, we generated a functional goat model of SND by ablating the SAN at the SEA. Our data show that following successful ablation, subsidiary pacemaker regions (SAP) emerge as the leading pacemaker, and the demonstrated characteristics of these pacemakers share features with human SND; they are inferiorly located within the RA, relatively bradycardic, show increased overdrive suppression, and demonstrate sinus pauses.

Radiofrequency ablation of the SAN is a complex procedure with poor outcomes in humans; in patients with inappropriate sinus tachycardia, the clinical recurrence rate was between 23 and 70% following SAN ablation (Lee et al., 1995; Man et al., 2000; Marrouche et al., 2002; Shen, 2002). As described above, the SAN is an extensive structure, which would require a large area to be ablated. In addition, in humans, the SAN is epicardial, so it’s protected from the endocardial application of radiofrequency energy heating effect by a layer of atrial myocardium as well as by the heat sink effect of high blood flow within the central SAN artery (Lee et al., 1995; Yokokawa et al., 2012). If the SAN is successfully ablated, the RA can be driven by a SAP at a slower cycle length (Matsuo et al., 2010). SAP tissue is functionally distinct from the SAN and it gives rise to a slower resting and exertional HR (Ardell et al., 1991). Over time, these differences subside and the SAP pacemaker becomes functionally more similar to the native SAN (Rozanski et al., 1984; Kalman et al., 1995).

However, classic annotation at the voltage maxima has shown to bias activation detection from the atrial tissue and not the smaller intramural structure of the SAN (Parameswaran et al., 2020; Yamabe and Orita, 2020). Both in clinical studies and in optical mapping systems of human atria, SAN activation has been detected by a positioned bipolar catheter as a low-amplitude deflection preceding the sharp atrial upstroke (Gomes et al., 1982; Li et al., 2017). Since the SEA represents the origin of the depolarization wavefront, it should precede any local electrogram and needs to be premature to the onset of the P-wave. In our work, we considered the SEA as the most premature local activation. In fact, the distal bipole showed a premature signal to both the intracardiac reference as well as the onset of the P-wave (Supplementary Material 3). It has been also suggested that SAN pathways would contain transitionary tissues (Csepe et al., 2016), which could be mechanistically explained by the presence of discrete pacemaker clusters within the SAN connecting to the surrounding atrial myocardium *via* specialized sinoatrial conduction pathways. Ablation, neuro-hormonal functional suppression, or dysfunction of superior conduction pathways would result in different atrial activation patterns *via* inferior conduction pathways (Li et al., 2017, 2020). It might be possible that activation mapping from the endocardial or epicardial surface could potentially miss the small potentials generated by the SAN prior to exit and capture of the atrial myocardium. This indeed may partly explain the low clinical success rates in SAN ablation. Despite the plurality of mechanisms, we have excluded the conducted regions by directly mapping the supposed SEA regions.

In our experiments, the acute endpoint of 50% reduction in HR or a pacemaker shift away from the epicardially accessible RA was successfully achieved during the radiofrequency ablation procedure in all cases (Supplementary Table 5). In all experimental goats, after the ablation procedure, the HR recovered promptly (Figure 7). Following this, over the 4 weeks follow-up period, a significant HR reduction was seen in those goats in which the ablation procedure was successful, but not in those in which the SAN ablation had failed. These functional data suggest that in goats, by silencing the SAN, a new leading SAP tissue can take over as the dominant pacemaker. In six experimental goats with successful ablation, five SAP tissues clustered along the caudal part of the CT adjacent to the IVC. These data would confirm previous similar findings in rat and dog, as well as being similar to the leading pacemaker site in humans with SND (Rozanski et al., 1984; Sanders et al., 2004; Morris et al., 2013).

Subsidiary pacemaker tissue was also functionally evaluated by a change in P-wave morphology and PR-interval, which was not detectable in those animals where the ablation procedure had failed. There was a significant shortening of the PR-interval in the SAP group as previously demonstrated in a canine model of SND (Kim et al., 1986). Further analysis of the P-wave before and after the ablation showed profound changes immediately after reaching the endpoint of procedure I. In fact, P-wave duration was significantly prolonged (*p* < 0.001, *n* = 10) in paired *T*-test (Supplementary Material 10). In addition, an inversion of the first portion of the P-wave appeared on the post-ablated ECG traces. A proper evaluation of any caudal–cranial switch activation pattern would have required precordial leads for accessing to the 12-lead ECG. In fact, no such changes in P-wave morphology in SND patients have been described yet. However, the pacemaker shift both in animal models and in healthy humans has been widely demonstrated to show P-wave inversion according to the location of the leading pacemaker (Boineau et al., 1988; Boyett et al., 2000; Monfredi et al., 2010). Moreover, in refractory inappropriate tachycardia patients, aside from the decrease in sinus rate, a typical endpoint during SAN ablation procedure is the inversion of the P-wave axis (Jacobson et al., 2014; Rodriguez-Manero et al., 2017). According to our results, the interatrial conduction time (defined by the first component of the PR-interval) had shortened with the emergence of the SAP, which was in closer distance to the atrioventricular node (Supplementary Material 11). Historically, the Bachmann’s bundle represents the main interatrial accessory for impulse-conducting pathways. Delay in this pathway may lead to either prolongation of the P-wave or interatrial block. More recently, catheter ablation technology and also the ability of treating specific arrhythmic targets with micro-anatomical precision have shown that, in addition to Bachmann’s bundle, other interatrial muscular bundles exist on the inferior atrial surface in close distance to the coronary sinus and also posteriorly in proximity of right upper and lower pulmonary veins (Ho et al., 1999; Chauvin et al., 2000; Kozłowski et al., 2002; Platonov et al., 2002; Mitrofanova et al., 2005). Furthermore, in previous works we showed that, in rat hearts, there is an extensive area in continuation of the SAN head toward the dorsal interatrial groove that appears HCN4 and Cx45 positive, suggesting that the cells in this region may have pacemaker activity (Yamamoto et al., 2006). Together with the SAN, it forms an inverted U-shaped atrial pacemaker complex. It was demonstrated that the isolation of SAN from the interatrial groove did not prevent this region to beat, hence proving that the SEA directly originated from within and its capability as a SAP (Yamamoto et al., 2006). In the goat heart, we confirmed the presence of a very similar inverted U-shaped atrial pacemaker complex, consisting of the compact SAN (located next to the CT) and the PNA area at both of its ends (Supplementary Material 12).

Previous works have showed that the new SAP tissue, immediately after the ablation procedure, not only reduces HR but is also readily suppressed by the overdrive pacing (Randall et al., 1982). We observed that the recovery time in the SAP group was longer than in the failed ablation group (rSN), suggesting that the degree of pacemaker suppression was greater in the SND model than in rSN group (Figures 8A,B). A prolonged recovery time of the SAN is indeed one diagnostic test for SND (Narula et al., 1972). In addition, the implantable loop recorder analyses showed in SAP goats the presence of transient junctional bradycardia and sinus pauses.

### The Heart Rate and the Autonomic Drive

Intrinsic properties of the SAN and the autonomic nervous system modulate HR. Although sympathetic and parasympathetic components were not directly investigated in this manuscript, we did not observe a significant autonomic drive in this context. As also reported in previous studies (Reimann et al., 1972; Hydbring et al., 1999), our data showed that the HR mean in the conscious vs. anesthetized animals was 87 ± 20 bpm and 84 ± 15 bpm (*n* = 15), respectively. Although HR mean did not vary significantly, HR variation revealed interesting differences. Under isoflurane, the beat- to- beat rate was uniform with a narrow deviation around the mean HR. By contrast, HR recorded in conscious animals showed a more dispersed distribution (Supplementary Material 13). General anesthesia depresses both cardiac parasympathetic and sympathetic drives resulting in a decreased HR variability (Vicenzi et al., 1995; Toader et al., 2011). ECGs in conscious animals were recorded in the presence of human operators, who might affect profoundly (observer effect) the autonomic balance of each individual goat (Kovacs et al., 2014). In the first post-operative 48 h, ECG recordings were unsuitable for valid analysis since the animals required artificial pacing for stabilizing rhythm. After the goats became independent from the pacemaker, HR was also evaluated by telemetry to observe HR changes over the course of 4 weeks. By comparing telemetry data with 4-min ECG recordings, we performed a linear regression analysis: unlike in the goats with an intact SAN, no strong correlation between HR assessed under general anesthesia was found. However, the 4-min conscious HR and the pacemaker telemetry data showed a significant correlation (Supplementary Material 14). Since no change in HR between anesthetized successfully ablated animals before and after 4 weeks procedure I was observed, we interpreted these findings as a SAP development over time as leading pacemaker. Lack of such a “maturation” of SAP tissue would lead to a significant lower heart rate in anesthetized animals, where parasympathetic and sympathetic components are abolished by general anesthesia. Also, we detected a significant tendency in reducing HR over time in conscious ablated animals.

### Insights Into Mechanisms for the Failure of Sinus Node Ablation

In six goats, the SAN was successfully ablated (described above). The remaining four goats had their SEA in an area identical to where all the pre-ablation leading pacemakers had been situated indicating ablation failure. In the rSN group, surviving islands of the SAN tissue were found on histological analysis (Figure 6C) and regions were found that showed a defined diastolic depolarization (Figure 6B). The aim of these observations was not to identify the “leading pacemaker” site of the SAN but just an interrogation of the ablated area for spontaneous action potentials. It was previously shown that moving away from the leading pacemaker, nodal action potentials present hyperpolarized membrane potentials, a longer cycle length, and change in action potential duration in transitional tissue and especially in the presence of “peripheral nodal cells” (Boyett et al., 1999, 2000). This would possibly explain the morphological features of the action potentials in our recording. Functionally, these small regions of surviving tissue were able to provide normal physiological pacemaker function with normal heart rate and minimal overdrive suppression (Figure 8).

The total ablated area within each experimental animal did not change, but we observed that in the failed ablated goats (rSN), the ablation time was greater when compared to the successful ablation (SAP) (Supplementary Material 5). We did not have force-sensing capability in the ablation catheter, so it may be that in the rSN group, the initial ablation lesions were ineffective and subsequent radiofrequency delivery was inhibited by tissue edema (Kolandaivelu et al., 2010; Yokokawa et al., 2011). In ablated animals, the SAN appeared completely and clearly fibrotic in histological sections. On the other hand, sections at the caudal level revealed that there was no change in the PNA structure between control and ablated goats (Supplementary Material 6). In this work, we were able to map the SEA in nine out of ten animals. In one goat, no signal was found to be premature both to the intracardiac reference electrode and to the onset of the P-wave on the surface ECG. This event could be explained by a new SEA, which emerged from the inter-atrial septum or from the left atrium (LA), which we were unable to map, as we did not have access to the LA.

## Study Limitations

Detailed investigation of the molecular architecture at mRNA level was limited by availability of primers for goat tissue and at protein level due to lack of specific antibodies.

In order to comply ethical and animal welfare, control animals were euthanized after locating the SEA in the SAN (procedure I). Thus, there has been no possibility to compare the follow-up in successful ablated animals with the controls.

In our microelectrode experiment, the simple superfusion was not strictly physiological. Thus, it would probably lead to transmural ischemia and increase in spontaneous beating cycle length. However, presented data aimed to show pacemaker potentials from within the ablated area of “failed ablation” animals to demonstrate qualitatively that there could be surviving, functioning pacemaker cells.

In this study, we could not rule out a change in SAN exit pathways; this would be impossible to do without optical mapping. However, a change in HR would be unlikely if it were only the exit to be ablated rather than the nodal tissue. Moreover, even though we directly mapped the SEA and not the conducted regions from the exit pathways, we could not exclude that small SAN potentials have not been missed. However, it is less likely with epicardial mapping than endocardial mapping.

## Conclusion

In this study, we generated a detailed description of the SAN region in goats and generated a model of SND in a large animal by ablating the SAN by applying radiofrequency energy. Moreover, the model allowed to map not only the SAN but also the PNA and the SAP tissues. Through histological and IF imaging, we revealed the presence of “nodal-like” clusters within the CT. These nodal clusters might have a key role as subsidiary pacemakers in pathophysiological conditions, becoming the dominant cardiac pacemaker. In addition, by expressing a mixture of an intermediate pattern of currents within the CT, similar to those typical of the SAN or RA, the SAP tissues might be prone to ectopic activity (Kalman et al., 1998; Tellez et al., 2006). Our model provides insight into the reasons for the difficulty and frequent failure of SAN ablation in humans. Indeed, in some goats, we demonstrated the persistence of small islands of SAN tissue, which were sufficient to support continued normal SAN function.

## Data Availability Statement

The raw data supporting the conclusions of this article will be made available by the authors, without undue reservation.

## Ethics Statement

The animal study was reviewed and approved by the University of Manchester Animal Welfare and Ethical Review Body in accordance with the “Animals (Scientific procedures) Act, 1986.” Written informed consent was obtained from the owners for the participation of their animals in this study.

## Author Contributions

LS: planning and manuscript writing of manuscript; re-analyzing data, creating, formatting and modifying figures and illustrations; discussion with senior authors (HD and GM) on format of manuscript and figures; incorporating all authors comments into final draft; and submitting manuscript. ZB: planning and execution of large animals’ sinus node ablation and subsidiary pacemaker mapping; carry out all *in vivo* electrophysiology and all *ex vivo* histology and immunohistochemistry experiments; analyzing all data and performing statistics; and producing initial figures and initiating writing of manuscript. JC: co-PI on funding; supervising ZB to carry out *in vivo* experiments; and contributing to analysis of data. BP, AV, AH, and YS: helping to plan and/or carry out *in vivo* electrophysiology experiments. YS, JY, and AA: helping with histology and proteins experiments. SL: carry out *ex vivo* electrophysiology experiments. BB: helping with initial draft of manuscript and figures. CG: holding licence for large animal research; co-PI on funding; supervising ZB with vivo experiments. GM: contributing to planning and writing of manuscript, re-analyzing, and formatting of figures and illustrations. HD: conceiving research; PI on funding; supervising ZB, AA, JY, and SL; and contribute to planning and writing of manuscript, re-analyzing, formatting of figures and illustrations. All authors approved the manuscript and data supplement.

## Conflict of Interest

The authors declare that the research was conducted in the absence of any commercial or financial relationships that could be construed as a potential conflict of interest.

## Footnotes

1 https://biorender.com/
