# Supplementary material for: Structural and Functional Properties of Subsidiary Atrial Pacemakers in a Goat Model of Sinus Node Disease
Source: Front Physiol. 2021 Mar 4;12:592229. doi: 10.3389/fphys.2021.592229 (PMC7969524; doi:10.3389/fphys.2021.592229)
Supplement: Supplementary file 1 [file Data_Sheet_1.PDF]

Supplementary Materials

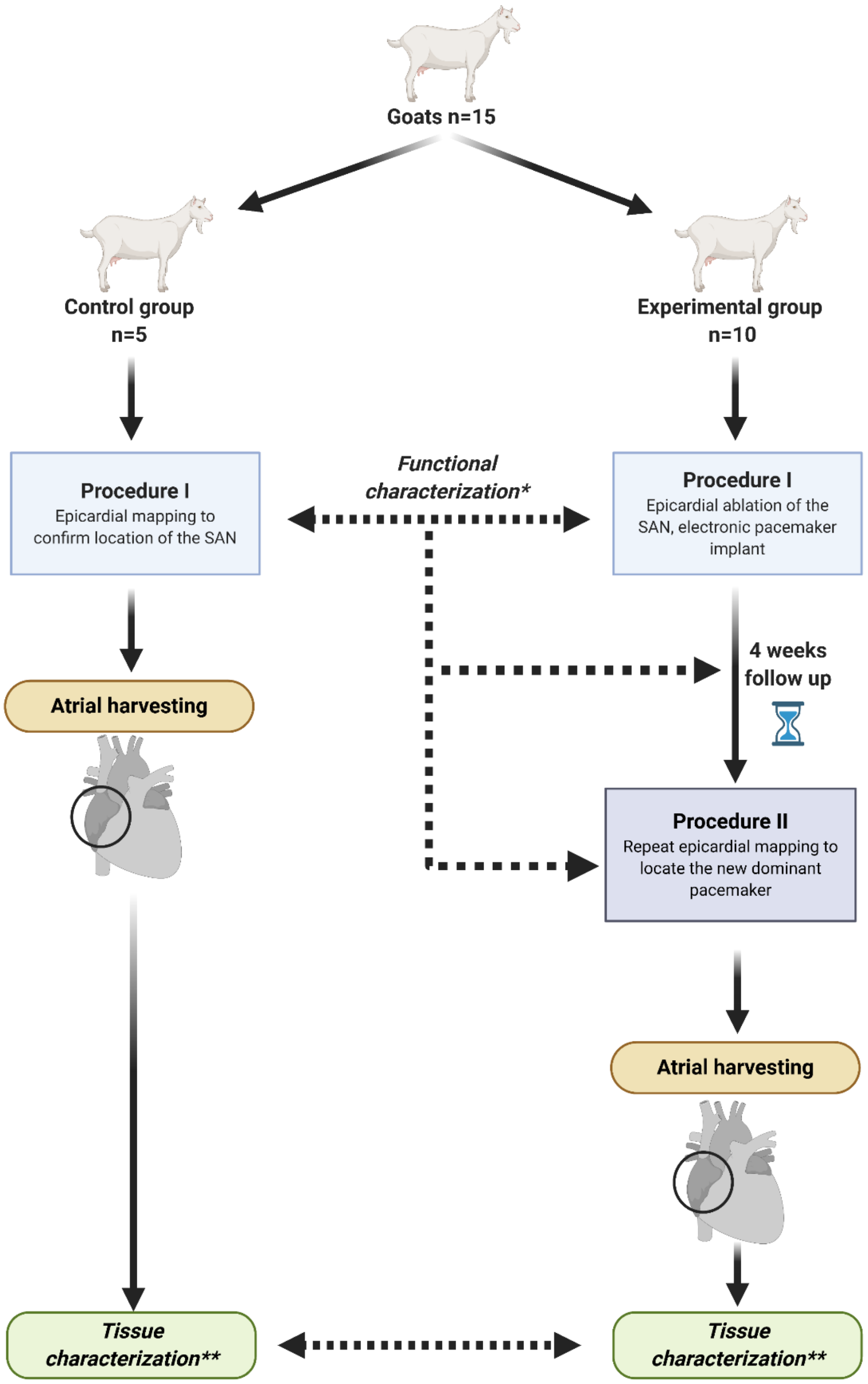

SM 1. Flow chart of the research protocol

*Surface ECG to assess heart rate, atrioventricular conduction time and P-wave morphology as well as assessment of SAN/SAP recovery time following overdrive pacing.

**Histology, qualitative and semi-quantitative immunohistochemistry were used, and characteristics of the SAN, working atrial myocardium and the newly emerging atrial pacemaker were compared.

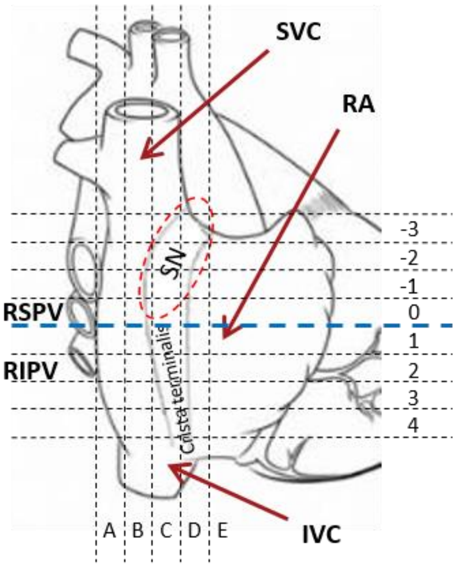

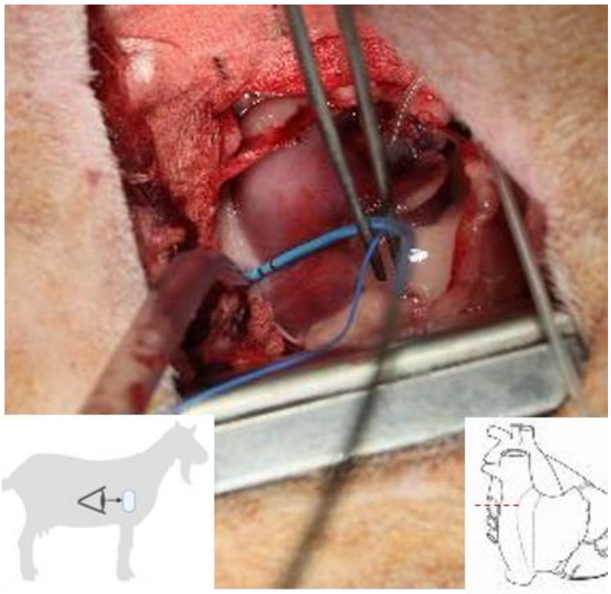

SM2. Epicardial grid to aid mapping

Left panel: the mapping catheter was placed in each area defined by the depicted virtual grid. The line of 0 (blue dashed line) represents the level of the right superior pulmonary vein (RSPV). From this point horizontal lines are drawn cranial and caudal with 5mm spacing corresponding to the inter-electrode distance of the mapping catheter. RIPV: Right inferior pulmonary vein. *Right panel*: mapping catheter placed at the line of zero. Inserts show the orientation.

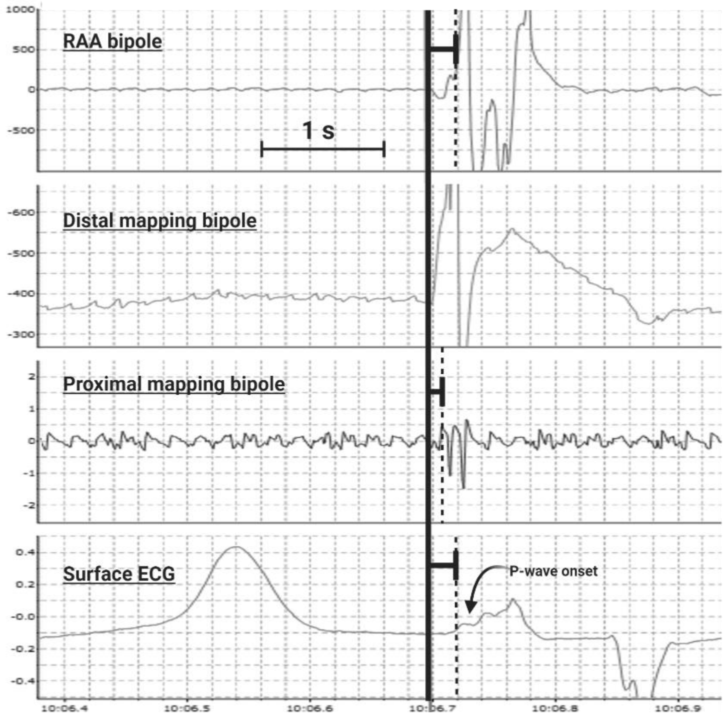

SM3. Mapping the site of earliest activation (SEA)

The distal bipole of the mapping catheter records the local activation time, which is premature to both the intracardiac reference (RAA) as well as to the onset of P-wave. The degree of prematurity is illustrated with the horizontal black lines.

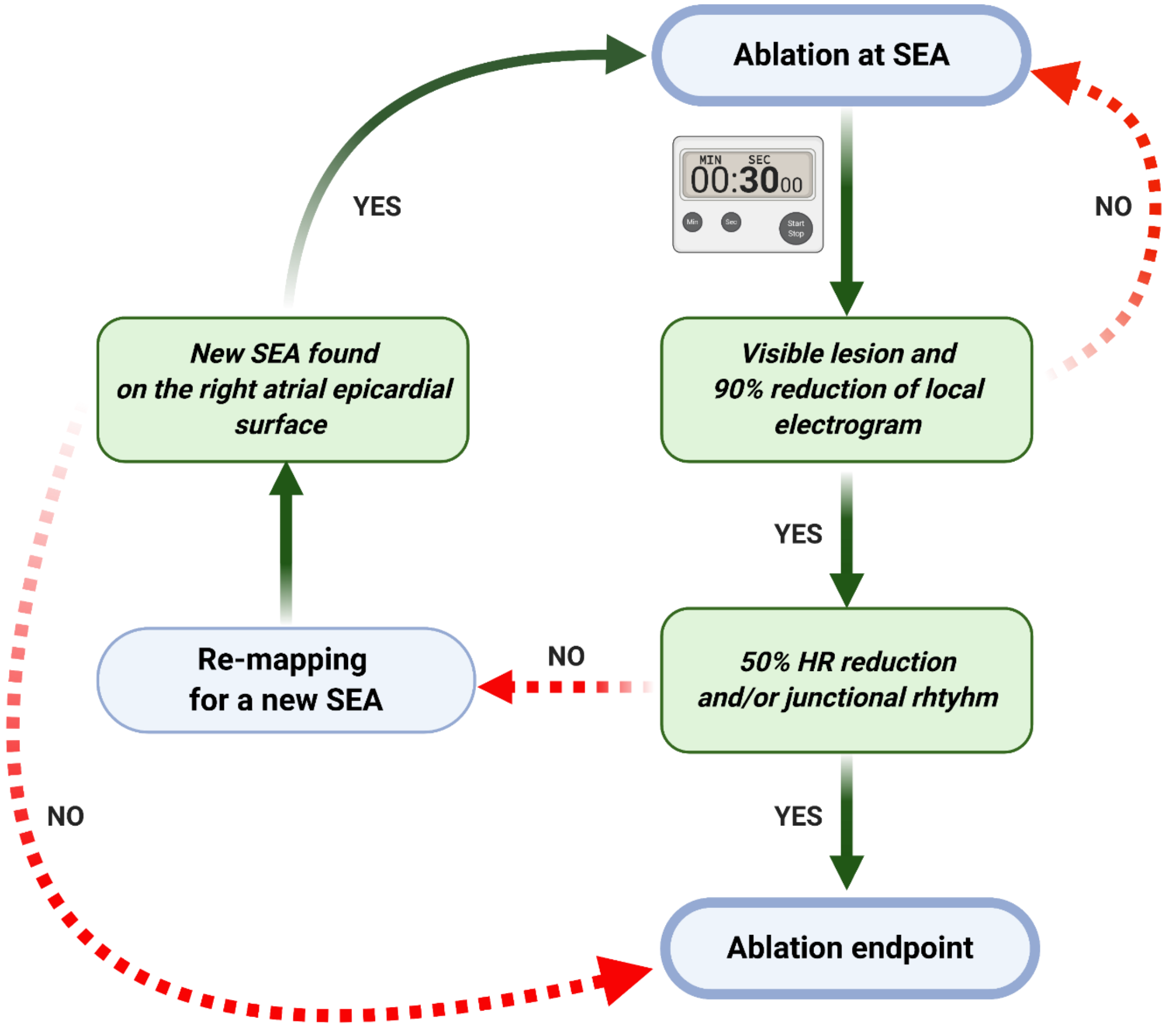

SM 4. Flow chart of the epicardial total SAN ablation.
If the new site of earliest activation (SEA) shifted away from the right atrial free wall to a site inaccessible to epicardial mapping (*i.e.* a location in the interatrial septum or the left atrium) there was no possibility of mapping.

Primary Antibodies					
Target	***Source***	***Isotype***	***Class***	***Manufacturer***	***Product n.***
**HCN4**	rabbit	IgG	polyclonal	*Alomone Labs*	APC-052
**NCX1**	mouse	IgM	monoclonal	*Thermo Scientific*	MA3-926
**Cx43**	rabbit	IgG	polyclonal	*Sigma*	C6219
**Cx43**	mouse	IgG	monoclonal	*Millipore*	MAB 3068
**HCN4**	rabbit	IgG	polyclonal	*Millipore*	MAB 5808

Supplementary table 1.
List of commercial primary antibodies used.

Secondary antibodies					
Target	***Source***	***Fluorochrome***	***Conjugate***	***Manufacturer***	***Product n.***
**Mouse IgG**	donkey	Cy3	polyclonal	*Millipore*	AP192C
**Mouse IgM**	donkey	FITC	polyclonal	*Sigma*	F9259
**Rabbit IgG**	donkey	FITC	polyclonal	*Millipore*	AP182F

Supplementary table 2.
List of commercial secondary antibodies used.

Fluorochrome	Absorption peak	Emission peak
FITC	492 nm	520 nm
Cy3	550 nm	570 nm

Supplementary table 3.
Properties of fluorochromes used. FITC (fluorescein isothiocyanate), Cy3 (indocarbocyanine).

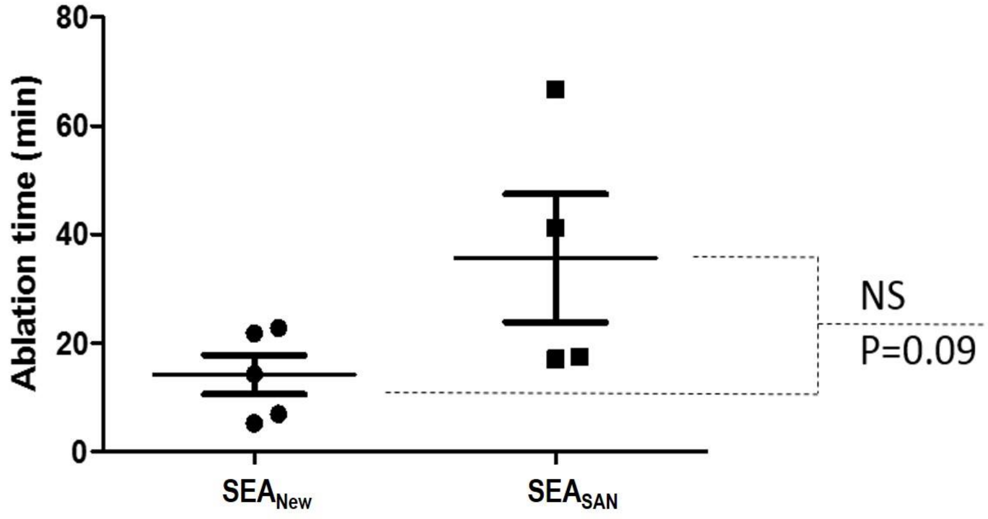

SM5. Total ablation time and success of SAN ablation

Goats with an emerging new SEA (SEA$_{new}$) showed a tendency in shortening the ablation time when compared to those with SEA within SAN (SEA$_{SAN}$) in unpaired T-test (SEA$_{new}$ 14.16 ± 3.64 min, n=5 vs. SEA$_{SAN}$ 35.60 ± 11.83 min, n=4, p=0.09). Excessive ablation may have caused tissue oedema, preventing effective power delivery.

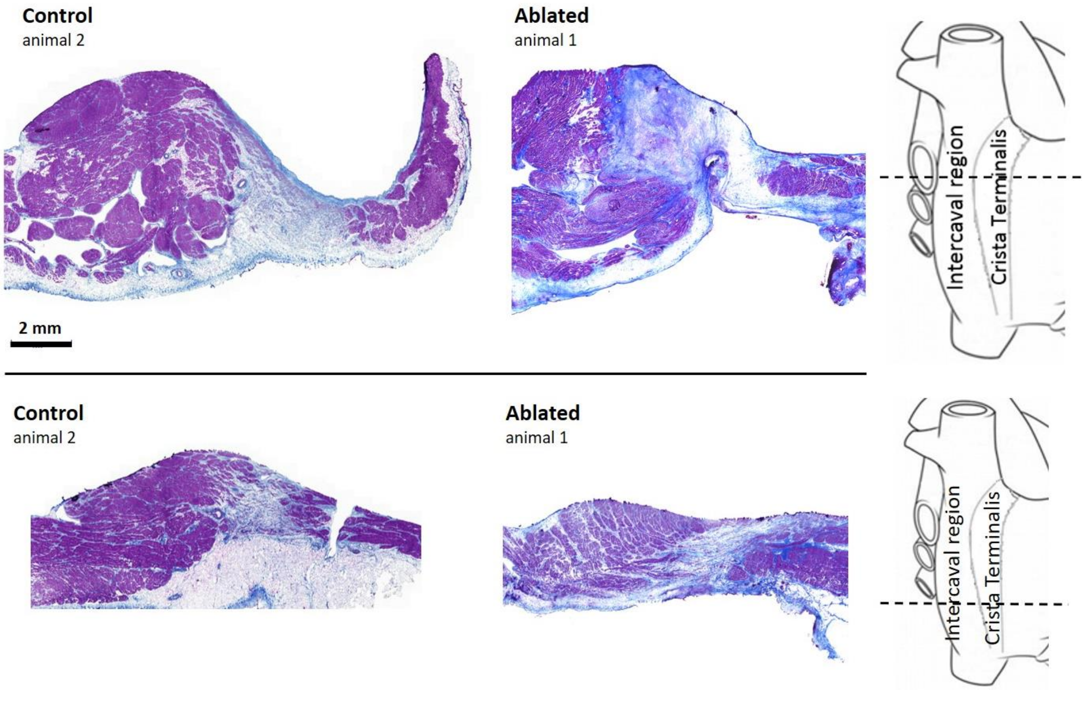

SM6. Cranial and caudal sections of sinus node in control and ablated goats

Upper histological images show major differences in fibrosis (blue stain) between control (small purple stained nodal cells) and ablated SN (mainly blue fibrotic tissue) SAN tissue. Lower images show both in control and experimental (ablated) animals interdigitations (*witchy fingers*) within the paranodal area. In ablated animals, this region (preserved from ablation) was not affected by fibrotic processes.

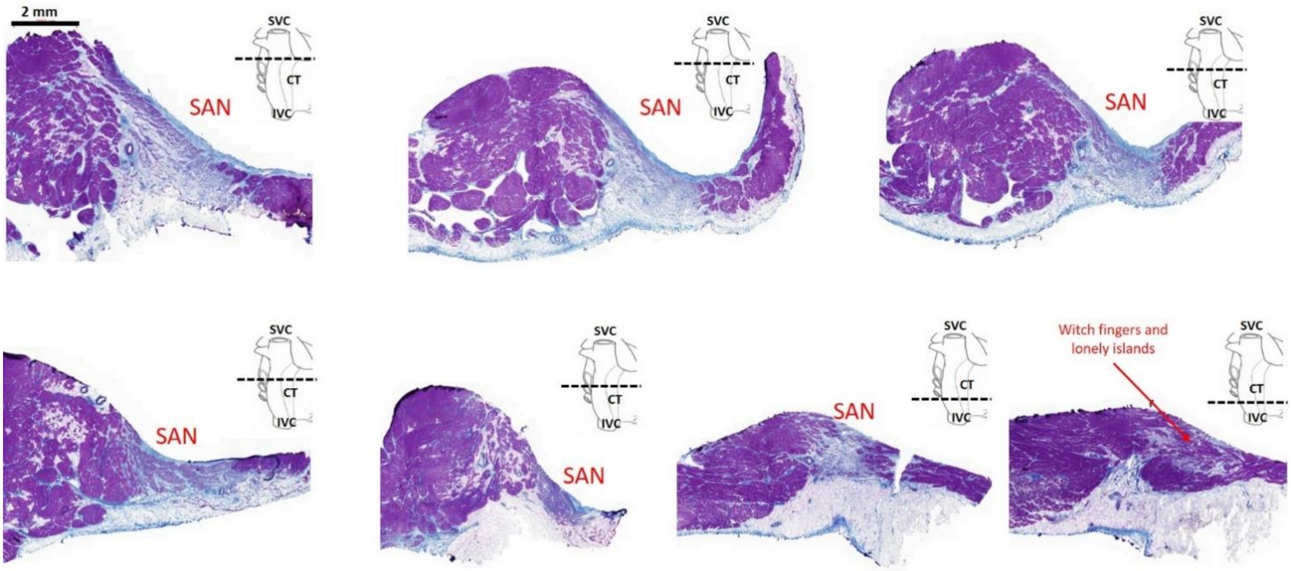

SM7. Cranial-Caudal sectioning of goat SAN

Histological representation of SAN location, within each section, in a control goat from the SVC towards IVC. In this species, we mainly observe the SAN being composed of the body (top panels) and tail (the bottom two panels on the left) and the last two bottom panels are nodal cells within the so-called PNA area. Masson's trichrome stained tissue section reveals pacemaker tissue, which stains paler when compared with working myocardium.

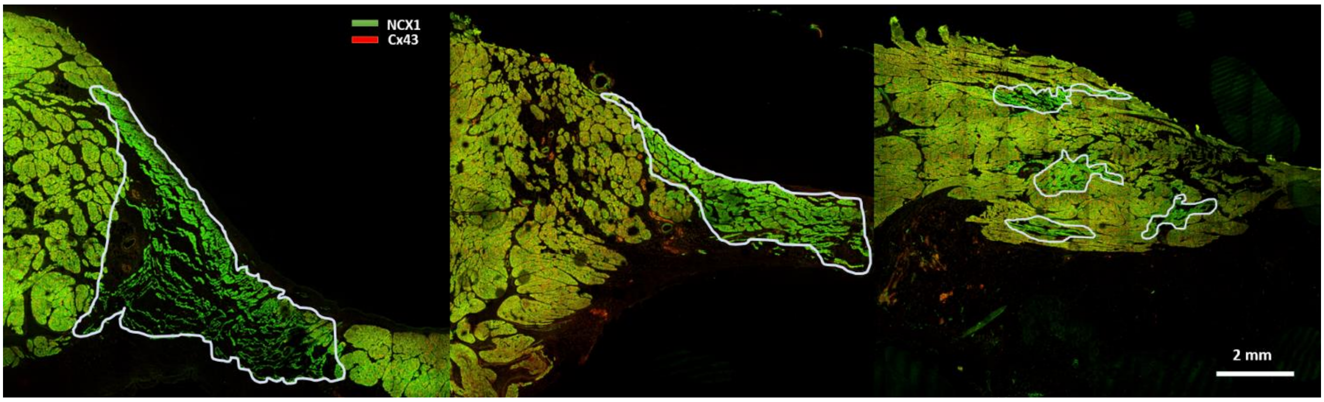

SM8. NCX1 and Cx43 protein expression in goat right atrium surrounding nodal tissue only positive for NCX1 expression

IF images shown here were selected adjacent to those shown in Figure 1D panel. NCX1 positive nodal tissue is highlighted in different sections.

	**RA**	± *SEM*	**SAN**	± *SEM*	**PNA**	± *SEM*	**RA**	± *SEM*	**SAP**	± *SEM*
HCN4	10.67	0.64	23.28	1.57	17.12	1.33	12.27	1.04	21.07	2.67
Cx43	19.89	1.85	12.05	0.50	15.27	0.28	20.02	1.94	17.67	3.16
NCX1	16.27	0.97	33.64	2.97	27.76	2.73	20.52	1.42	32.27	1.00

Supplementary table 4. IF data of protein expression profile from **Figure 2** are reported as mean ± SEM. Semi-quantitative analysis of HCN4, Cx43 and NCX1 expression (based on IF signal intensity profile) was conducted on experimental (n=4) vs. control (n=4) animals (total, n=8). High-power images were digitalised at 10-bit colour depth and analysed through Volocity software, which measured the sum of pixel intensities.

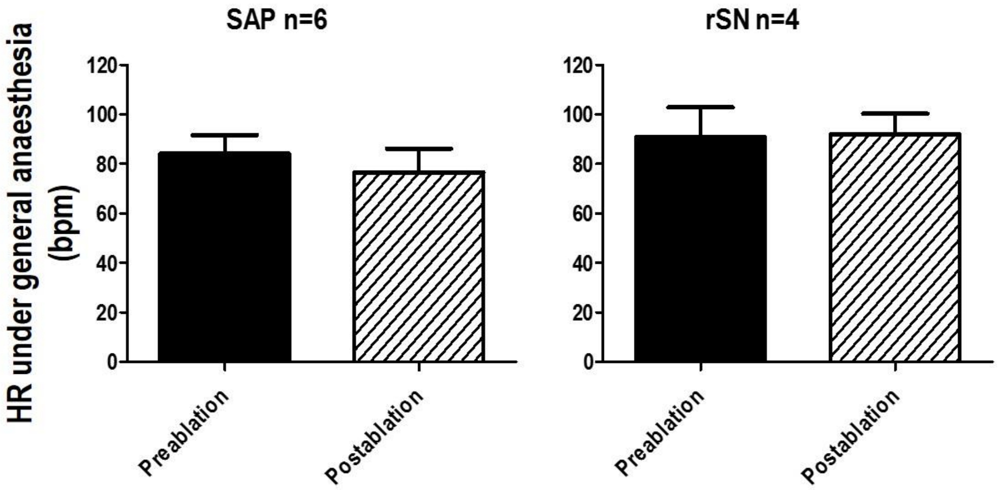

SM9. Heart rate comparison *pre*- and *post*- SAN ablation

HR under general anaesthesia prior to ablation and at the end of the 4 weeks. SAP and rSN goats do not show significant differences in HR.

Goats (n=10)	Reduction in HR (%)	post ablation rhythm	endpoint
1	67	junctional	>50% HR
2	59	junctional	>50% HR
3	50	atrial	>50% HR
4	52	atrial	>50% HR
5	36	atrial	pacemaker shift
6	100	none	>50% HR
7	32	atrial	pacemaker shift
8	53	atrial	>50% HR
9	69	atrial	>50% HR
10	50	atrial	>50% HR

Supplementary table 5. Acute outcome in the experimental group following SAN ablation
Predefined endpoint was achieved in all cases. In five cases, ablation was ended when 50% rate drop was observed with atrial escape rhythm. In three cases no atrial escape rhythm was observed. In two occasions, only modest heart rate reduction was achieved due to a pacemaker shift away from the right atrial free wall where further mapping and ablation was not possible (animals 5 and 7).

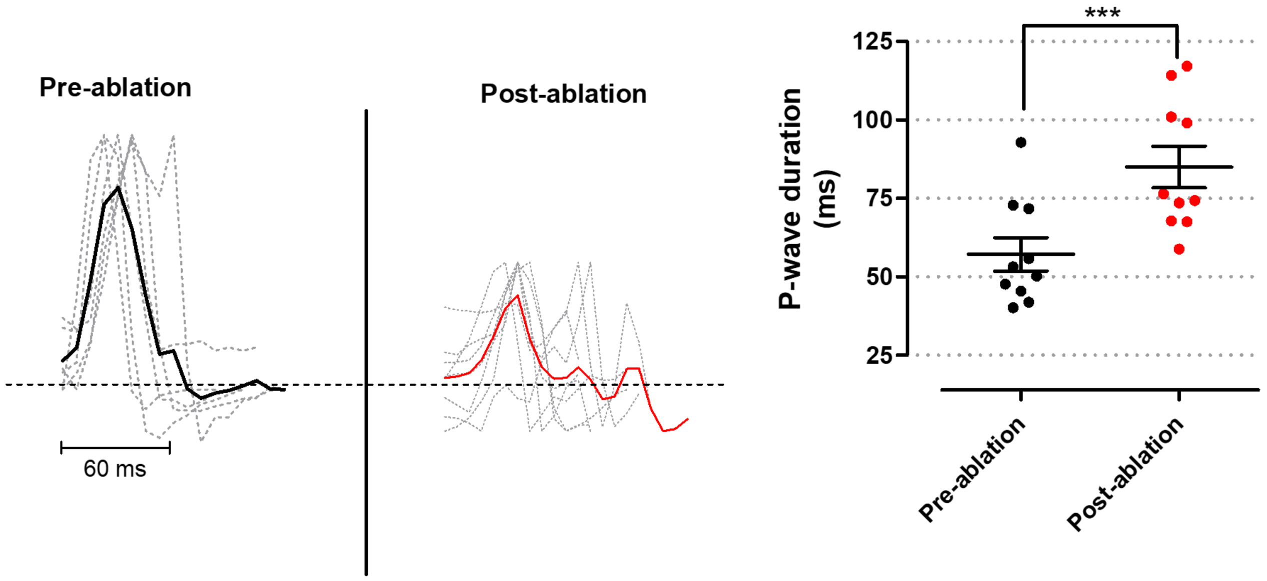

SM6 10. P-wave morphology and duration
The first panel shows P-wave morphology before and after the ablation procedure (reached endpoint). For each single goat, up to n=30 P-waves (not shown) were averaged and measured for their duration (ms). Highlighted traces represent averaged P-waves from each goat (n=10). The second, P-wave duration before and after the ablation procedure. After ablation P-wave duration is significantly prolonged (*pre*-abl 57.2 ± 5.3 ms vs. *post*-abl 84.9 ± 6.6 ms, p<0.001, n=10). Two-tailed paired T-test has been applied. Data are expressed as mean value ± SE.

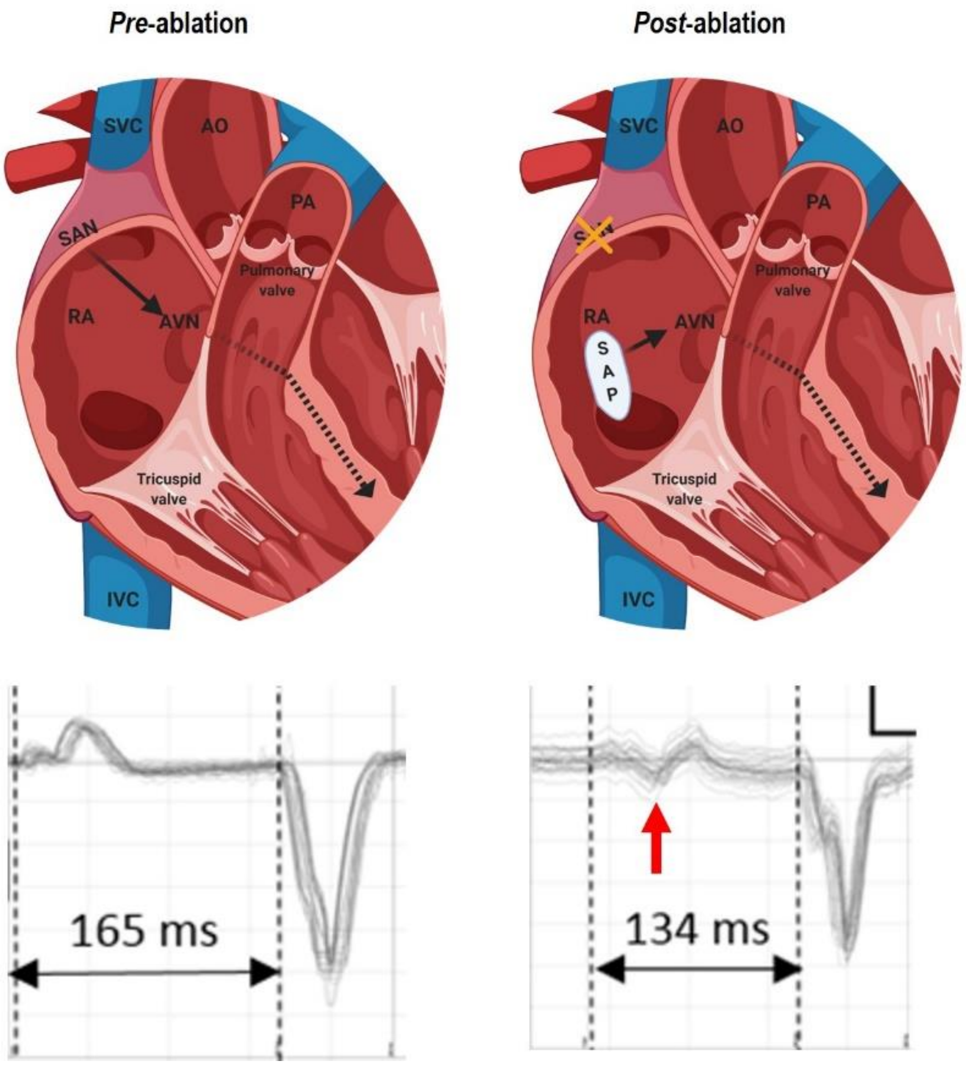

SM11. Inter-atrial component of the P-wave
The schematic diagram shows the morphological change of the first component of the P-wave according to the location of the leading pacemaker. After ablating the SAN, the electrical impulse is generated from the SAP tissue and reaches the atrioventricular node in a shorter time than from the SAN. Moreover, the direction shift of the electrical impulse towards the atrioventricular node generates a change in the inter-atrial component of the P-wave morphology (red arrow) detectable on the ECG. *Aorta* (AO); *atrioventricular node* (AVN); *inferior vena cava* (IVC); *pulmonary artery* (PA); *sinoatrial node* (SAN); *subsidiary atrial pacemaker* (SAP); *superior vena cava* (SVC); *right atrium* (RA).

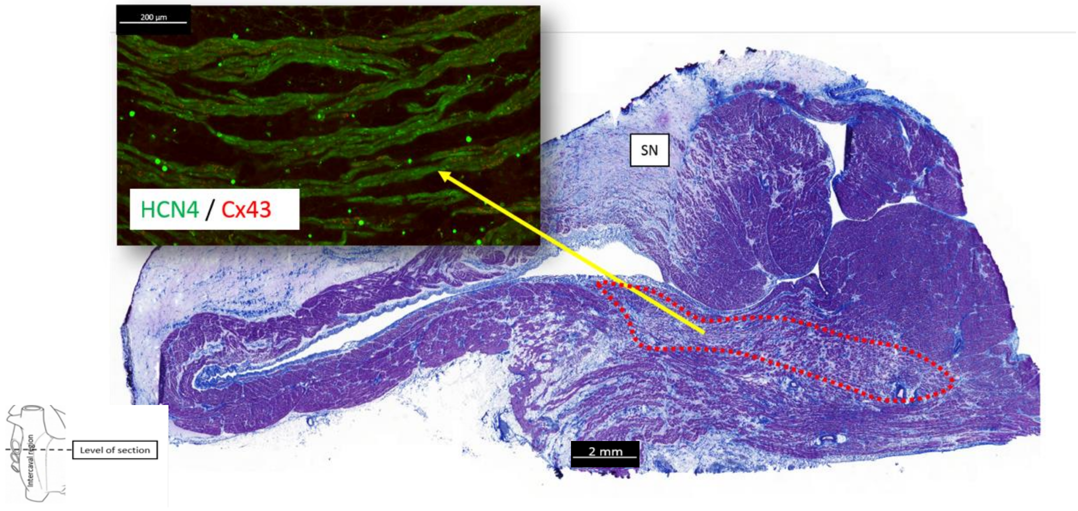

SM12. HCN4 positive nodal-like cells in the second PNA

A histological section and its orientation (inset, bottom left) was taken from a control animal. An IF slide (adjacent to the histology slide, inset, top left) reveals a mixture of $HCN4^{+}/Cx43^{+}$ positive region from the area of the second 'PNA' within the atrial septum. In this control specimen, the RA and SVC are intact and confirmed that the SAN continues downward into the interatrial groove and is converted into a second 'PNA' area (red outline). Unlike the nodal-like cells, which are paler and smaller than the atrial cells, the second 'PNA' is darker stained and expresses a mixture of Cx43 and HCN4. This second 'PNA' area was only collected from one goat and should be investigated in more animals in the future.

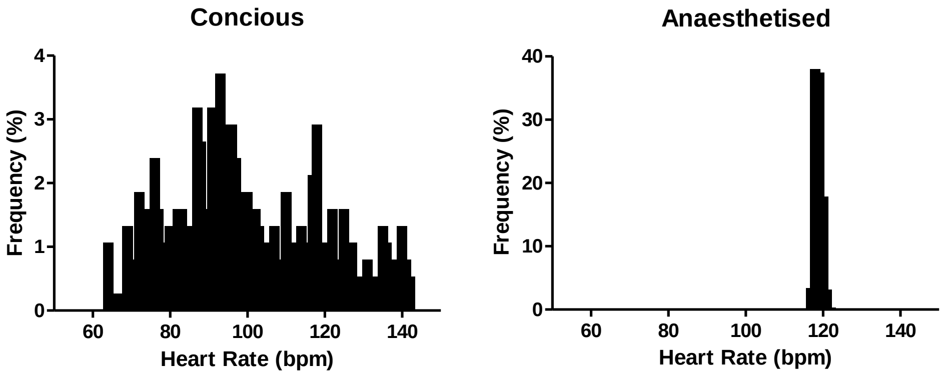

SM913. Heart rate variance in conscious vs. anaesthetised goats

Heart rate histogram computed from continuous 4 min ECG in the same goat, same day prior to ablation. The conscious recording (left) has a much wider *beat-to-beat* variation compared to the measurements taken under general anaesthesia. The dispersion of data points in the conscious goats is visually obvious and would be confirmed by a markedly higher standard deviation.

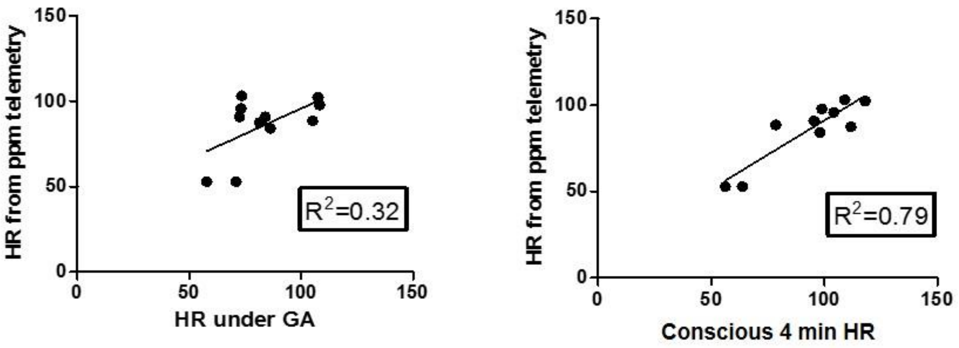

SM14. HR correlation in the SN ablated state

The HR was assessed post-ablation. Correlation was found only between the *short term* vs. *long term* HR, measured in conscious animals (R^2=0.79, p<0.001).
